# The impact of study design and diagnostic approach in a large multi-centre ADHD study: Part 2: Dimensional measures of psychopathology and intelligence

**DOI:** 10.1186/1471-244X-11-55

**Published:** 2011-04-07

**Authors:** Ueli C Müller, Philip Asherson, Tobias Banaschewski, Jan K Buitelaar, Richard P Ebstein, Jaques Eisenberg, Michael Gill, Iris Manor, Ana Miranda, Robert D Oades, Herbert Roeyers, Aribert Rothenberger, Joseph A Sergeant, Edmund JS Sonuga-Barke, Margaret Thompson, Stephen V Faraone, Hans-Christoph Steinhausen

**Affiliations:** 1 Department of Child and Adolescent Psychiatry, University of Zurich, Zurich, Switzerland; 2 Departement Pädagogisch-Therapeutische Berufe, Hochschule für Heilpädagogik, Zurich, Switzerland; 3 MRC Social Genetic Developmental and Psychiatry Centre, Institute of Psychiatry, London, UK; 4 Department of Child and Adolescent Psychiatry and Psychotherapy, Central Institute of Mental Health, J 5, Mannheim, Germany; 5 Department of Psychiatry, Radboud University Nijmegen Medical Center, Nijmegen, The Netherlands; 6 Department of Psychology, Hebrew University, Jerusalem, Israel; 7 Department of Psychiatry, School of Medicine, Trinity College Dublin, Dublin, Ireland; 8 Geha MHC, Petach-Tikva, Israel; 9 Department of Developmental and Educational Psychology, University of Valencia, Valencia, Spain; 10 Clinic for Child and Adolescent Psychiatry and Psychotherapy, University of Duisburg-Essen, Essen, Germany; 11 Department of Experimental Clinical and Health Psychology, Ghent University, Ghent, Belgium; 12 Department of Child and Adolescent Psychiatry, University of Göttingen, Göttingen, Germany; 13 Department of Clinical Neuropsychology, Vrije Universiteit, Amsterdam, The Netherlands; 14 School of Psychology, University of Southampton, Southampton, UK; 15 Departments of Psychiatry and of Neuroscience and Physiology, SUNY Upstate Medical University, Syracuse, NY, USA; 16 Aalborg Psychiatric Hospital, Aarhus University Hospital, Aalborg, Denmark; 17 Clinical Psychology and Epidemiology, Institute of Psychology, University of Basel, Basel, Switzerland

**Keywords:** ADHD, multi-centre study, sibling design, centre effects

## Abstract

**Background:**

The International Multi-centre ADHD Genetics (IMAGE) project with 11 participating centres from 7 European countries and Israel has collected a large behavioural and genetic database for present and future research. Behavioural data were collected from 1068 probands with ADHD and 1446 unselected siblings. The aim was to describe and analyse questionnaire data and IQ measures from all probands and siblings. In particular, to investigate the influence of age, gender, family status (proband vs. sibling), informant, and centres on sample homogeneity in psychopathological measures.

**Methods:**

Conners' Questionnaires, Strengths and Difficulties Questionnaires, and Wechsler Intelligence Scores were used to describe the phenotype of the sample. Data were analysed by use of robust statistical multi-way procedures.

**Results:**

Besides main effects of age, gender, informant, and centre, there were considerable interaction effects on questionnaire data. The larger differences between probands and siblings at home than at school may reflect contrast effects in the parents. Furthermore, there were marked gender by status effects on the ADHD symptom ratings with girls scoring one standard deviation higher than boys in the proband sample but lower than boys in the siblings sample. The multi-centre design is another important source of heterogeneity, particularly in the interaction with the family status. To a large extent the centres differed from each other with regard to differences between proband and sibling scores.

**Conclusions:**

When ADHD probands are diagnosed by use of fixed symptom counts, the severity of the disorder in the proband sample may markedly differ between boys and girls and across age, particularly in samples with a large age range. A multi-centre design carries the risk of considerable phenotypic differences between centres and, consequently, of additional heterogeneity of the sample even if standardized diagnostic procedures are used. These possible sources of variance should be counteracted in genetic analyses either by using age and gender adjusted diagnostic procedures and regional normative data or by adjusting for design artefacts by use of covariate statistics, by eliminating outliers, or by other methods suitable for reducing heterogeneity.

## Background

Attention Deficit Hyperactivity Disorder (ADHD), one of the most prevalent disorders in childhood and adolescence, is characterized by problems in allocating attention, regulating motor activity, and controlling behavioural impulses [1]. In many subjects, the disorder is accompanied by comorbid conditions including conduct disorders, oppositional defiant disorders, mood disorders, and anxiety disorders [2]. Furthermore, intellectual abilities are often impaired in children with ADHD [3]. The disorder may affect not only all aspects of a child's life, including familial functioning, but also often persists into adulthood [1,4].

The risk for having ADHD is 2 to 8-fold higher in parents of children with ADHD than in the normal population and is elevated in siblings of children with ADHD [5]. These findings indicate a strong familiality of the disorder. Twin and adoption studies have frequently reported a heritability for ADHD of about 75% [1,6,7]. Quite often, siblings of ADHD children are subjected to an intermediate level of the disorder that lies between that shown by the affected probands and the healthy controls without a diagnosis of ADHD, e.g. with respect to ADHD symptomatology [5], comorbid conditions [8,9], intellectual abilities [10-12], or cognitive tasks performance [13].

The complexity of ADHD, not only in terms of the clinical picture, but also of the underlying pathophysiology and causes [1] implies that identified causal 'units', e.g. single genes, or single environmental factors, have only a small effect on the risk of developing ADHD [14]. Therefore, the investigation of the causes of ADHD needs large and homogeneous samples in order to have the power that is needed for the detection of etiological sources with small effects.

The International Multicentre ADHD Genetics (IMAGE) project [14-16] provides a large database for molecular genetic investigations of ADHD. This database contains behavioural data from almost 1400 European Families with one child affected by ADHD, and one or several unselected siblings. Additionally the DNA of all participants is stored in a cell line repository, enabling almost infinite numbers of molecular genetic ADHD studies in the future.

The recruiting and assessment procedure, described in detail in the companion paper [17], included screening with the use of questionnaires, checking for inclusion/exclusion criteria, procedures for verifying the ADHD diagnosis, ratings from teacher and parent questionnaires, IQ measurement, and collection of DNA by blood samples or mouth swabs. Inclusion criteria were Caucasian ethnicity; one child with a probable diagnosis of ADHD of the combined type; at least one sibling, regardless of ADHD symptoms; DNA available from at least four genetic family members, including the proband with ADHD, at least one sibling, and at least one parent; and the age of the children lying between five and seventeen years. Exclusion criteria were IQ<70 in the children, a diagnosis of schizophrenia or autism, including atypical autism; a neurological disorder of the central nervous system, or a genetic disorder that might mimic ADHD. The diagnoses of all probands and of the siblings suspected to have ADHD were then verified using a diagnostic interview with the parents in combination with a symptom checklist generated from a teacher questionnaire. Siblings fulfilling the criteria of ADHD were excluded from the sibling sample. The questionnaires were completed by both the parents and the teachers, except for the questionnaire assessing autistic symptoms, which was completed only by the parents. A short form of an IQ test was applied by trained clinicians. An overview of studies of the IMAGE project published so far is available in the companion paper of the present contribution [17] or at the periodically updated IMAGE homepage http://image.iop.kcl.ac.uk.

The present paper aims to describe and analyse the behavioural phenotype of the IMAGE sample consisting of 1068 probands and 1446 siblings. In contrast to the companion paper [17], which analysed the symptom-based diagnostic characteristics of 1068 probands and 339 siblings, a dimensional approach is chosen in the present paper. Influences of age, gender, family status (proband vs. sibling), informant (parents vs. teacher), and study-centre on questionnaire scores and intelligence (IQ) measures are analysed using robust multi-way procedures. This report focuses on the degree of psychopathological heterogeneity caused by these factors and by characteristics of the measures applied and their underlying normative samples. In the first part of this study [17] we argued, that diagnostic criteria based on a defined number of symptoms can mask age- or gender-related distortions in the sample structure, particularly in the associated genotypic structure. Similarly, the present second part deals with the question of whether and how the questionnaire and IQ findings are biased due to the study design and diagnostic procedures applied.

The behavioural measures used in the IMAGE project reflect its main purpose of providing a large database for molecular genetic studies. Intelligence is associated with ADHD and may also be an endophenotype of ADHD [12]. Intelligence quotient (IQ) measures should be assessed and considered as possible covariates in statistical analyses. The Conners' questionnaires are validated instruments for the assessment of ADHD [18,19]. A symptom checklist as well as dimensional scores can be derived from these questionnaires. Additionally, they include scores for the most common comorbid conditions of ADHD. The Strengths and Difficulties Questionnaire (SDQ) is another widely used instrument for the assessment of ADHD and comorbidities including emotional problems, conduct problems, and peer problems [20]. Furthermore, a score measuring prosocial behaviour can be derived from the SDQ. This score in combination with the Social Communications Questionnaire [21], is used in screening for autism spectrum disorders as autistic features are frequently associated with ADHD [22,23].

The interpretation of the results must bear in mind to which of three categories the data belong. These categories reflect the nature and degree of standardisation applied to the data and lead to different expectations about the effects of independent factors on the data. One category comprises the IQ measures. These result from a direct assessment of the child's abilities, and the raw scores are translated into standardized scores that take age into account. In addition, it should be noted that different normative samples are used for each language. A second category comprises the standardized questionnaire measures reflecting the parents' and teachers' perceptions of the behaviour of the child. These scores are age and gender specific, but, in contrast to the IQ measures, are based on a single normative sample across all centres. The third category comprises all non-standardized questionnaire scores (raw scores) reflecting the parents' and teachers' perceptions of the behaviour of the child without formally considering age, gender, language, or other demographic factors.

Depending on the characteristics of each category of data, in theory different effects would be expected. Measures belonging to the first category (IQ) would be expected to reveal gender effects, but no effects of age and study-centre, assuming that socio-cultural differences are reflected in the language-specific normative samples. Measures of the second category (the standardized questionnaire scores) would be expected to be free of age and gender effects, but probably not of study-centre effects, because only one (US) normative sample is used. Measures of the third category (the non-standardized questionnaire scores) would be expected to reveal age, gender, and study-centre effects. It is important to consider that all three predictions concern theoretical assumptions based on a population of unaffected children.

Consequently, we expect our sample to deviate from these assumptions because ADHD is not considered to depend linearly on changes in age, gender and other effects [24-26].

In all three categories, we expect to find clear differences in the effect of the family status between probands and siblings in almost all variables, because the siblings of children with ADHD are known to be affected more strongly than healthy controls but less severely than their brothers and sisters with ADHD (see above). These effects may be overlaid with so called contrast effects in the parent ratings leading to a relative overestimation of symptoms in the probands compared to their siblings and vice versa [27,28].

Furthermore, based on our symptom-based analyses of the IMAGE sample [17] we expect to find considerable of study-centre. Because these study-centre effects were also found between centres in the same countries, we decided to define centres and not countries as recruiting units in the analysis in both papers.

## Methods

### Participants

The sample for the present analyses consisted of 1068 probands (938 boys and 130 girls) aged 5 - 17 years with a DSM-IV [29] diagnosis of Attention Deficit Hyperactivity Disorder, Combined Type (ADHD-CT), 1446 unselected siblings (730 boys and 716 girls) in the same age range, and their parents. The participating families were recruited within the International Multi-centre ADHD Genetics (IMAGE) project with 11 participating centres from 7 European countries and Israel, namely Amsterdam (NLD_A), Dublin (IRL_D), Essen (GER_E), Gent (BEL_G), Göttingen (GER_G), Jerusalem (ISR_J), London and Southampton (ENG_L/S), Nijmegen (NLD_N), Petah Tiqva (ISR_P), Valencia (ESP_V), and Zürich (SWI_Z) between April 2003 and April 2007.

The diagnosis was based on both the Parental Account of Childhood Symptom (PACS) [30-33] and the teacher form of the Conners' questionnaires (CTRS:R-L) [34]. For a more detailed description of the sample, the study design, the inclusion criteria, and the diagnostic protocol see part 1 of this contribution [17].

### Measures

The children's behaviour was assessed by teacher and parent forms of the Conners' questionnaire (CTRS:R-L and CPRS:R-L) [34], the Strengths and Difficulties Questionnaire (SDQ) [20], and by the parent form of the Social Communication Questionnaire (SCQ) [21].

The parent version of the Conners' questionnaires, the CPRS-R:L [34], contains 80 questions and the teacher version, the CTRS-R:L [35], contains 59 questions which are grouped into the following 14 scales: (1) oppositional, (2) cognitive problems/inattention, (3) hyperactivity, (4) anxious/shy, (5) perfectionism, (6) social problems, (7) psychosomatic, (8) Conners' ADHD index, (9) Conners' global index: emotional lability, (10) Conners' global index: impulsivity, (11) Conners' global index: total, (12) DSM-IV ADHD symptoms: inattention, (13) DSM-IV ADHD symptoms: hyperactivity/impulsivity, and (14) DSM-IV ADHD symptoms: total. In the present study, standardized scores (T-scores) based on the US normative sample were used [36].

The Strength and Difficulties Questionnaire SDQ [20] comprises 25 questions and allows computation of raw scores for the following five scales: emotional symptoms, conduct problems, hyperactivity (and inattention), peer problems, and prosocial behaviour.

The Social Communication Questionnaire SCQ [21] contains 40 questions dealing with autism spectrum disorder symptoms. The number of positively answered questions adds up to a total score with a cut-off value of 14 for autism spectrum disorder and 21 for classical autism.

In addition to the behavioural assessments, intelligence was assessed with the WISC-III [37] (age<17) or the WAIS-III [38](age> = 17). The following subtests were assessed: vocabulary, similarities, block design, picture completion, and digit span. Scaled scores of each subtest were calculated using validated versions of the WISC/WAIS according to the language of the test person. The intelligence quotient (IQ) was prorated from two verbal subtests (vocabulary and similarities) and two performance subtests (picture completion and block design) using an algorithm based on correlations among the subtests [39]. Digit span was chosen as a measure of working memory.

### Statistical procedures

The distributions of the data in the samples and subsamples deviated markedly from normality and symmetry and the subsamples had unequal variances and sample sizes, as emphasized in part I [17]. Moreover, comparisons between subsamples (e.g. probands vs. siblings) were often skewed in opposite direction. Therefore, in the present contribution we applied methods which are robust to deviations from normality, symmetry, equal sample sizes, and homogeneity of variance. The following statistical procedures were used:

- The percentile bootstrap procedure *trimpb *[40,41], with 2000 bootstrap samples, was applied to compute robust

 confidence intervals for means and trimmed means in R [42].

- Robust three-way analyses were calculated in R [42] by applying the procedure *t3way *[41,43], a heteroscedastic method for trimmed means with estimates of standard errors and degrees of freedom adjusted for the amount of trimming, unequal variances and unequal sample sizes. This method provides a test value ('Q') which can be used to test null-hypotheses of main effects and interactions and adjusted critical values ('crit.') for the 1-alpha quantile of a chi-square distribution.

- Robust post-hoc pairwise comparisons were computed in R [42] by using the bootstrap procedure *  linconb6 *[44], an expansion of the procedure *lincon *[43], which allows unequal variances; 599 bootstrap samples were taken by default; familywise 95% confidence intervals, corresponding to a 5% probability of making at least one Type I error when performing multiple tests, were calculated.

- The adapted robust 'between × between × within ANOVA' procedure *bbtwin *[41,44] was applied to compare two dependent groups (parents and teacher ratings) when including two dichotomous covariates (gender and family status) with respect to 20% trimmed means.

- The residuals of linear regression analyses on age [45] were used instead of raw scores in order to adjust statistics for age effects.

- Effect sizes are reported in units of standard deviations, calculated by converting the T-scores (Conners' questionnaires), the prorated IQ, and the standard scores of the IQ subscales, or by use of the scores of a British normative sample in the case of the SDQ [46].

## Results

### Conners questionnaires

Conners' questionnaire data were available from 1068 probands with ADHD-CT and their 1446 unselected siblings. The male to female ratios were 7.2:1 for the probands and 1.0:1 for the siblings (for a detailed analysis of demographic data, see the companion paper [17].

Table S1 (additional file 1) shows quartiles with 95% confidence intervals of trimmed population means for all Conners' scales, divided by informant, gender, and family status. Overlaid histograms of the sample distributions for each scale of the Conners' questionnaires, divided by family status and informant, are displayed in Figure S1 (additional file 2).

Although the T-scores for the Conners' subscales are adjusted for age (and gender), there were small, but significant correlations between age and almost all Conners' T-scores, both in the parents' (average rho = .06) and in the teachers' ratings (average rho = .10; see Table 1). The three-way analyses of centre-, status-, and gender effects, therefore, were performed on the basis of age corrected scores (residuals of the scores' linear regression on age).

**Table 1:** Conners' Questionnaires: Effects of age, centre, status, and gender

**Parent ratings**																							
	
	***Age***		***Centre1°***			***Status*°**			***Gender*°**			***Centre × Status*°**			***Centre × Gender*°**			***Status × Gender*°**			***Centre × Status × Gender*°**		
								
	***rho***	***p***	***Q***	***Crit***	***Sig***	***Q***	***Crit***	***Sig***	***Q***	***Crit***	***Sig***	***Q***	***Crit***	***Sig***	***Q***	***Crit***	***Sig***	***Q***	***Crit***	***Sig***	***Q***	***Crit***	***Sig***
								
***A***	0.063	0.002	69.2	22.9	***	595.9	4.01	***	0.55	4.01		103	22.9	***	17.9	22.9		16.5	4.01	***	22.0	22.9	
***B***	0.002	0.919	13.8	22.7		881.2	4.05	***	28.7	4.05	***	81.4	22.7	***	6.87	22.7		70.3	4.05	***	10.6	22.7	
***C***	0.099	0.000	12.3	20.8		2001	3.94	***	1.71	3.94		69.0	20.8	***	8.43	20.8		53.1	3.94	***	14.3	20.8	
***D***	0.076	0.001	16.8	25.0		82.1	4.05	***	3.84	4.05		42.5	25.0	**	8.42	25.0		14.6	4.05	***	11.8	25.0	
***E***	-0.073	0.000	168.0	23.8	***	76.06	4.11	***	1.74	4.11		32.8	23.8	**	14.1	23.8		1.67	4.11		17.7	23.8	
***F***	-0.002	0.938	31.4	24.7	*	371.3	4.03	***	6.41	4.03	*	43.0	24.7	**	24.0	24.7		15.6	4.03	***	26.6	24.7	*
***G***	0.020	0.312	21.6	24.7		69.78	4.03	***	8.75	4.03	**	12.4	24.7		14.2	24.7		6.22	4.03	*	6.69	24.7	
***H***	0.053	0.008	32.3	20.7	**	1327	4.00	***	19.5	4.00	***	109	20.7	***	9.5	20.7		90.6	4.00	***	20.5	20.7	
***I***	0.080	0.000	17.1	21.3		1343	3.99	***	4.94	3.99	*	95.3	21.3	***	3.08	21.3		59.0	3.99	***	7.79	21.3	
***J***	0.125	0.000	65.7	23.4	***	299.7	4.11	***	0.44	4.11		79.4	23.4	***	15.7	23.4		12.9	4.11	**	18.5	23.4	
***K***	0.099	0.000	17.8	21.8		1133	4.00	***	1.68	4.00		104	21.8	***	6.29	21.8		50.2	4.00	***	11.8	21.8	
***L***	0.045	0.027	15.9	22.3		1057	3.98	***	27.8	3.98	***	108	22.3	***	6.95	22.3		78.0	3.98	***	15.3	22.3	
***M***	0.072	0.000	14.8	21.1		1850	3.96	***	3.90	3.96		66.1	21.1	***	6.79	21.1		46.7	3.96	***	11.2	21.1	
***N***	0.070	0.001	10.0	20.6		1823	3.95	***	17.5	3.95	***	101	20.6	***	8.14	20.6		84.9	3.95	***	16.9	20.6	
							
***Mean ^§^***	0.0628		36.2	22.6		922.1	4.0		9.1	4.0		74.7	22.6		10.7	22.6		42.9	4.0		15.1	22.6	
																							
**Teacher ratings**																							
	
	***Age***		***Centre*°**			***Status*°**			***Gender*°**			***Centre × Status*°**			***Centre × Gender*°**			***Status × Gender*°**			***Centre × Status × Gender*°**		
								
	***rho***	***p***	***Q***	***Crit***	***Sig***	***Q***	***Crit***	***Sig***	***Q***	***Crit***	***Sig***	***Q***	***Crit***	***Sig***	***Q***	***Crit***	***Sig***	***Q***	***Crit***	***Sig***	***Q***	***Crit***	***Sig***
								
***A***	0.060	0.004	85.5	25.1	***	159.7	4.22	***	0.68	4.22		19.5	25.1		8.28	25.1		4.90	4.22	*	11.2	25.1	
***B***	0.122	0.000	55.2	23.8	***	235.1	3.97	***	9.94	3.97	**	12.1	23.8		16.3	23.8		18.4	3.97	***	10.5	23.8	
***C***	0.114	0.000	32.9	22.1	**	560.0	3.96	***	33.7	3.96	***	10.4	22.1		19.8	22.1		29.8	3.96	***	18.5	22.1	
***D***	0.151	0.000	30.3	26.1	*	43.42	4.27	***	1.06	4.27		4.6	26.1		5.82	26.1		1.64	4.27		9.6	26.1	
***E***	-0.024	0.254	104	22.2	***	39.09	4.02	***	6.82	4.02	*	8.8	22.2		9.66	22.2		1.54	4.02		6.90	22.2	
***F***	0.057	0.006	30.3	25.1	*	146.2	4.20	***	6.23	4.20	*	24.9	25.1		40.3	25.1	**	6.63	4.20	*	37.1	25.1	**
																							
***H***	0.134	0.000	74.0	21.3	***	647.7	3.95	***	30.9	3.95	***	8.8	21.3		24.97	21.3	*	44.3	3.95	***	16.4	21.3	
***I***	0.115	0.000	43.1	21.0	***	622.8	3.98	***	27.0	3.98	***	11.6	21.0		27.2	21.0	*	43.1	3.98	***	14.4	21.0	
***J***	0.087	0.000	48.3	22.7	***	224.2	3.96	***	0.01	3.96		36.5	22.7	**	51.4	22.7	***	2.58	3.96		59.4	22.7	***
***K***	0.102	0.000	59.4	21.0	***	618.4	3.94	***	14.1	3.94	***	11.5	21.0		27.6	21.0	*	30.1	3.94	***	17.6	21.0	
***L***	0.145	0.000	77.7	21.2	***	496.8	3.90	***	24.6	3.90	***	5.6	21.2		35.9	21.2	**	32.9	3.90	***	17.3	21.2	
***M***	0.083	0.000	34.2	21.4	**	639.1	3.93	***	39.1	3.93	***	11.2	21.4		13.0	21.4		38.6	3.93	***	16.4	21.4	
***N***	0.147	0.000	69.3	20.9	***	844.9	3.91	***	57.2	3.91	***	8.2	20.9		22.9	20.9	*	55.7	3.91	***	16.8	20.9	
							
***Mean ^§^***	0.0958		57.3	22.6		406.0	4.0		19.3	4.0		13.3	22.6		23.3	22.6		23.9	4.0		19.4	22.6	

***A ***Oppositional

***B ***Cognitive Problems/Inattention

***C ***Hyperactivity

***D ***Anxious-Shy

***E ***Perfectionism

***F ***Social Problems

***G ***Psychosomatic

***H ***Conners' ADHD-Index

***I ***Conners' Global Index: Restless-Impulsive

***J ***Conners' Global Index: Emotional Lability

***K ***Conners' Global Index: Total

***L ***DSM-IV: Inattentive

***M ***DSM-IV: Hyperactive-Impulsive

***N ***DSM-IV: Total

° Three-way analysis; dependent scores are adjusted for age (residuals of linear regression)

§ In age: mean of absolute 'rho' values

*Q *Robust three-way test statistic for trimmed means (see methods section)

Crit Critical value (α = .05) for Q

Sig Two sided α-level

* p < 0.05

** p < .0.01

*** p < .0.001

#### Status effects (siblings vs. probands)

**Figure 1:**
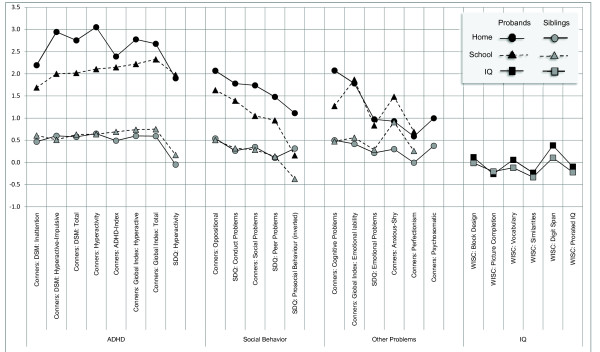
**Effect sizes of questionnaire scores and IQ measures, divided by family status and informant**. Notes: The indicated scores are positively correlated with symptom severity in questionnaires, and with intelligence in IQ scores, respectively. See methods sections for information about normative samples.

When looking globally at all 14 symptoms, there was a strong average effect of family status as evident in the difference between the teachers' average trimmed mean scores in probands (66.9) and in siblings (52.9), and even more strongly in the parents' ratings (70.8 in probands, 51.8 in siblings; see Figure 1 for effect sizes). Statistical three-way analyses of age-adjusted Conners' scores with gender, status, and centre as factors confirmed this average effect of family status: probands had higher scores than siblings in both parents' and teachers' ratings for all the symptoms (Table 1).

#### Family status by gender interactions

There was clear evidence for a gender by status interaction: male probands had lower average scores across scales (66.4) than female probands (73.1) based on both teacher and parent ratings (70,1 in probands, 76.9 in siblings). In contrast, male siblings had slightly higher average scores (53.6) than girls (52.1) for the teacher ratings and also higher scores (53.3) than girls (50.3) for the parent ratings.

These differential gender effects, dependent on family status, were statistically confirmed by highly significant gender by status interactions in the three-way analyses of symptom frequencies for almost all parents and teacher scores (see Table S1 in additional file 1). A closer look at the scales with a missing or low status by gender interaction shows that these scales (A, D, E, F, G, J) mainly assessed comorbid problems (social behaviour, anxiety, perfectionism, psychosomatic features). This finding indicates that higher symptom frequencies in girls compared to boys in the proband sample and vice versa in the sibling sample were mainly present in the ADHD-related scales.

#### Gender effects and centre by gender interactions

On average, boys had higher trimmed mean scores (61.4) than girls (55.0) in the teachers' ratings and even more pronounced in the parents' ratings (64.0 in boys, 53.8 in girls). Statistical three-way analyses on age adjusted questionnaire scores with gender, status, and centre as factors revealed gender effects for most of the scores. These effects were more pronounced for teacher ratings, as shown by the higher average Q statistics (19.3) compared to the parents average Q of 9.1 (Table 1). The scales without gender effects, after adjustments for age, family status, and centre, were 'oppositional behaviour' in both parents' and teachers' ratings, 'hyperactivity' in the parents' ratings, 'anxious-shy' in both ratings, and 'perfectionism', 'emotional lability', 'total global index', and 'DSM-IV: hyperactive' based on parent ratings. Gender effects were strong (Q>14, p < .001), particularly, in scales with one or more components of ADHD core symptoms (scales B, C, H, I, K, L, M). All of these scales had higher trimmed mean scores in boys than in girls (Table S1 in additional file 1).

There were no centre by gender interactions based on parent ratings indicating that the parents' perception of the similarity or diversity between ratings of boys and girls was equivalent across centres. In contrast, there were seven scales with a significant centre by gender effect based on teacher ratings including 'emotional lability', 'DSM: inattention', and 'social problems' with the highest significances (all p < .01).

#### Study-centre effects and centre by status interactions

Centre effects, after controlling for age, gender, and family status, were mainly present in the teacher ratings (Table 1; all effects are significant). In contrast, only five scores from the parent questionnaire differed between centres, namely 'oppositional', 'perfectionism', 'emotional lability' (all p < .001), 'social problems' (p < .05), and 'ADHD-index' (p < .01). In contrast to these stronger centre effects for the teacher ratings, centre by status interactions were almost exclusively seen in the parent ratings.

#### Post hoc pairwise comparisons between study-centres

Post hoc analyses of centre effects were calculated in the three ADHD DSM-IV scales (L, M, N) and the scale 'oppositional' (A). Because centre by status effects were significant in the parent scales, these analyses were conducted separately for probands and siblings. Figure S2 (additional file 3) shows trimmed mean scores for the four selected scales across all centres, separately for probands and siblings. The significant centre by status interaction is evident in the higher number of significant pair differences in probands compared to siblings (probability level adjusted for multiple tests). The numbers of significant differences (out of 55 in each scale) amounted to 11 (oppositional), 12 (DSM-IV: inattentive), 18 (DSM-IV: hyperactive), and 19 (DSM-IV: total) in probands, but only 5 (oppositional), 7 (DSM-IV: inattentive), 4 (DSM-IV: hyperactive), and 7 (DSM-IV: total) in siblings.

When the rank positions of the centres were compared across scores, some patterns became evident: ENG_L/S, IRL_D, and BEL_G had the highest scores on the three DSM-IV scales in the proband sample, whereas ISR_P and GER_G, had low scores on these scales in the same sample. In some centres, e.g. ESP_V, ISR_J, the rank positions were similar between the DSM_IV scales but they differed from the 'oppositional' scale. In the sibling sample, the discrepancy between the 'oppositional' scale and the DSM_IV scales seemed to be less pronounced. When the proband sample was compared to the sibling sample with respect to the centre rank positions, notable differences became evident. For instance, the probands from IRL_D had high relative scores on all scales, whereas the siblings from the same centre had low scores compared to the other centres; similarly, but in the inverse direction, the probands from ISR_P had low scores compared to other centres, whereas the siblings had the highest scores (Figure S2 in additional file 3).

Due to the absence of significant centre by status interaction effects in the teacher scales, post-hoc comparisons were conducted with the whole sample. These analyses resulted in 15 (oppositional), 8 (DSM-IV: inattentive), 6 (DSM-IV: hyperactive), and 5 (DSM-IV: total) significantly different pairs of study-centres (probability level adjusted for multiple tests; Figure S3 in additional file 4). The rank order of the centres with regard to mean scores was very stable across centres: BEL_G, ENG_L/S, NLD_A, and NLD_G had low scores, GER_G, SWI_Z, and IRL_D had medium scores, and ESP_V, GER_E, ISR_J, and ISR_P had high scores.

#### Informant effects and interactions

After controlling for age, status, and gender, parents and teachers differed in their ratings only on the scales 'oppositional', cognitive problems', and 'social problems' (Table 2). All mean scores were higher when rated by the parents compared to the teachers (Table S1 in additional file 1).

**Table 2:** Conners' Questionnaires: Effects of Informant (with gender and status)

	*Effects of gender and informant*°							
	**Informant**		**Status × Informant**		**Gender × Informant**		**Status × Gender × Informant**	
				
	***Q***	***P***	***Q***	***P***	***Q***	***p***	***Q***	***p***
***A***	7.909	0.005	11.428	0.001	0.050	0.823	0.745	0.388
***B***	12.200	0.000	116.045	0.000	1.458	0.228	16.505	0.000
***C***	0.001	0.970	75.375	0.000	19.397	0.000	0.495	0.482
***D***	1.190	0.276	1.767	0.184	1.173	0.279	2.029	0.155
***E***	0.436	0.509	6.123	0.014	0.017	0.896	1.694	0.193
***F***	6.333	0.012	38.783	0.000	0.530	0.467	2.896	0.089
***H***	0.004	0.949	15.906	0.000	2.979	0.085	0.698	0.404
***I***	0.217	0.641	41.139	0.000	6.117	0.014	0.000	0.989
***J***	1.391	0.239	0.035	0.852	0.004	0.947	0.429	0.513
***K***	0.002	0.967	15.127	0.000	2.110	0.147	0.025	0.874
***L***	3.218	0.073	70.426	0.000	0.146	0.702	8.148	0.004
***M***	0.050	0.823	43.202	0.000	15.097	0.000	1.631	0.202
***N***	0.207	0.649	75.474	0.000	6.687	0.010	1.400	0.237

**° **Results of robust between (status) by between (gender) by within (informant) ANOVA; dependent variables are adjusted for age; main effects of status and gender, and their interaction, are not shown

*Q *Q-statistic of robust between × between × within ANOVA

***A ***Oppositional

***B ***Cognitive Problems/Inattention

***C ***Hyperactivity

***D ***Anxious-Shy

***E ***Perfectionism

***F ***Social Problems

***H ***Conners' ADHD-Index

***I ***Conners' Global Index: Restless-Impulsive

***J ***Conners' Global Index: Emotional Lability

***K ***Conners' Global Index: Total

***L ***DSM-IV: Inattentive

***M ***DSM-IV: Hyperactive-Impulsive

***N ***DSM-IV: Total

However, there was a highly significant informant by status effect for all scales except the scale 'anxious-shy'. This interaction effect resulted from a general pattern present in almost all scales: there were higher parent ratings compared to teacher ratings in the proband sample (mean difference 4.4; see Table S1 in additional file 1), but similar or slightly lower parent ratings in the sibling sample (mean difference -1.0; see Figure 1 for effect sizes).

A gender by informant effect - after controlling for status and all remaining interactions - was present only in the four scales measuring 'hyperactivity', 'global index: restless-impulsive', 'DSM_IV: hyperactive-impulsive', and 'DSM_IV: total' with all containing a substantial hyperactivity component. This interaction effect resulted from the similar ratings by both informants in the female sample (difference from -2.1 to 0.9), but markedly higher parent ratings than teacher ratings in the male sample (differences from 4.3 to 8.7; see Table S1 in additional file 1). This finding indicates an informant effect for boys but not for girls for these four hyperactivity related subscales. Three-way interactions were only present in the 'cognitive problems' and 'DSM-inattentive' subscales.

### Strengths and difficulties questionnaire

#### Age effects

Correlations between age and SDQ scales were weak but significant for the 'hyperactivity' scale both for the parent ratings (rho = -.046) and the teacher ratings (rho = -.058). This finding points to a slight decrease of hyperactivity with age. Additionally, the 'emotional problems' scale was correlated positively with age for the teacher ratings (rho = .068) indicating an increase of emotional problems with age (Table 3).

**Table 3:** Strengths and Difficulties Questionnaire (SDQ) and Social Communication Questionnaire (SCQ) Effects of Centre, status, and gender (adjusted for age)

**Parent ratings**																							
	
	**Age**		**Centre°**			**Status°**			**Gender°**			***Centre × Status*°**			***Centre × Gender*°**			***Status × Gender*°**			***Centre × Status × Gender*°**		
								
	***rho***	***P***	***Q***	**Crit**	***Sig***	**Q**	**Crit**	***Sig***	**Q**	**Cri**	***Sig***	**Q**	**Crit**	***Sig***	**Q**	**.Cri**	**Sig**	**Q**	**Crit**	**Sig**	**Q**	**Crit**	**Sig**
								
***CP***	-0.023	0.265	44.6	24.3	***	344.1	4.04	***	8.06	4.04	**	36	24.3	**	12.4	24.3		10.0	4.04	**	10.2	24.3	
***EP***	0.020	0.334	19.6	23.4		122.8	3.98	***	1.0	3.98		27.0	23.4	*	15.70	23.4		2.6	3.98		29.8	23.4	*
***H***	-0.046	0.022	26.7	19.9	**	1653	3.89	***	58.90	3.89	***	151.3	19.9	***	15.52	19.9		40.6	3.89	***	14.9	19.9	
***PB(i)***	0.030	0.133	152	22.9	***	69.35	4.41	***	32.34	4.41	***	48.1	22.9	***	14.0	22.9		5.56	4.41	*	14.6	22.9	
***PP***	0.021	0.520	23.6	24.7		200.0	4.23	***	2.26	4.23		28.6	24.7	*	16.0	24.7		3.8	4.23		20.5	24.7	
***AvP***	-0.008	0.681	16.3	22.2		937.71	3.95	***	16.82	3.95	***	90.3	22.2	***	20.1	22.2		25.83	3.95	***	19.36	22.2	
***SCQ***	0.009	0.673	699.9	24.0	***	107.9	4.31	***	6.88	4.31	*	78	24.0	***	15.6	24.0		3.6	4.31		15.8	24.0	
																							
**Teacher ratings**																							
	
	**Age**		**Centre°**			**Status°**			**Gender°**			***Centre × Status*°**			***Centre × Gender*°**			***Status × Gender*°**			***Centre × Status × Gender*°**		
								
	***rho***	***P***	***Q***	**Crit**	***Sig***	**Q**	**Crit**	***Sig***	**Q**	**Cri**	***Sig***	**Q**	**Crit**	***Sig***	**Q**	**.Cri**	**Sig**	**Q**	**Crit**	**Sig**	**Q**	**Crit**	**Sig**
								
***CP***	-0.005	0.809	52.5	24.5	***	120.9	4.37	***	15.53	4.37	***	7	24.5		5.9	24.5		1.0	4.37		16.6	24.5	
***EP***	0.068	0.001	23.0	24.8		52.2	4.09	***	3.2	4.09		2.6	24.8		9.43	24.8		4.2	4.09	*	8.9	24.8	
***H***	-0.058	0.005	38.6	20.9	***	602	3.92	***	60.33	3.92	***	10.1	20.9		24.59	20.9	*	11.9	3.92	***	9.3	20.9	
***PB(i)***	0.038	0.062	53.1	22.7	***	95.2	4.01	***	40.64	4.01	***	21.1	22.7		19.80	22.7		2.8	4.01		7.4	22.7	
***PP***	0.028	0.175	41	23.8	**	127.33	4.18	***	1.60	4.18		9.9	23.8		14.4	23.8		1.43	4.18		13.8	23.8	
***AvP***	0.000	0.991	69.4	23.4	***	361.5	4.11	***	17.75	4.11	***	6.8	23.4		24.2	23.4	*	8.1	4.11	**	7.4	23.4	

*SDQ scales*

***CP ***Conduct Problems

***EP ***Emotional Problems

***H ***Hyperactivity

***PB(i) ***Prosocial Behaviour (inverted)

***PP ***Peer Problems

***AvP ***Average Problems

*SCQ scale*

***SCQ ***Total score

° Between-by-within design; dependent score is adjusted for age (residuals of linear regression)

*Q *Robust between/within test statistic for trimmed means (see methods section)

Crit. Critical value (α = .05)

Sig two sided α-level

* p < 0.05

** p < .0.01

*** p < .0.001

All of the following analyses were based on residuals of the scales on age (see methods), independent of the degree and significance of the correlation between the scales and age.

#### Average Problem scale

The distributions of the SDQ scales divided by gender, family status, and informant are displayed as histograms in Figure S4 (additional file 5) and as quartiles in Table S2 (additional file 6) with 20% trimmed means and their 95% confidence intervals.

The average problem scale (AvP; see Table A2 in additional file 6) composed out of the four problem scales conduct problems (CP), emotional problems (EP), hyperactivity (H), and peer problems (PP) showed higher average scores in the parent ratings (trimmed mean = 3.6) compared to the teacher ratings (3.0), and higher scores in boys compared to girls both for the parent (4.3 : 2.1) and the teacher ratings (3.5 : 1.9). As expected, the average problem scores for probands were also higher than the sibling scores both for the parent (5.2 : 2.1) and the teacher ratings (4.2 : 2.0).

These differences in the group means suggest that the main effects of gender, status, centre, and informant, and probably the interaction effects of gender by informant and status by informant were due to greater differences in the parent ratings compared to the teacher ratings.

#### Effects of family status (probands vs. siblings)

Statistical three-way analyses of age-corrected SDQ scores including gender, family status, and centre revealed strong family status effects in the four problem scales (CP, EP, H, PP) for the parent ratings (Table 3 Figure 1): Q statistics were between 122 and 1653 (5% critical values between 3.89 and 4.23, all p < .001). Similarly, all status effects based on teacher ratings were highly significant, but slightly smaller (Q from 52.2 to 602; critical values from 3.92 to 4.37). The average problem score summarised these problem effects and was clearly higher at home (Q = 937) than at school (Q = 127).

Table 3 demonstrates that the family status effect, as perceived by teachers and by parents, was by far the strongest for 'hyperactivity', somewhat weaker for 'conduct problems' and 'peer problems', and weakest for 'emotional problems'. The 'prosocial behaviour' ratings were also more problematic for probands than siblings; this status effect was weaker at home than at school.

#### Effects of gender

For both the parent and the teacher ratings, significant effects of gender were present in the two problem scales measuring 'conduct problems' and 'hyperactivity', in the strengths scale 'prosocial behaviour', and in the 'average problem scale', but not in the scales measuring 'emotional problems' and 'peer problems'. As demonstrated in Table S2 (additional file 6), all scores indicated greater problems in boys compared with girls. The gender effect was strongest for 'hyperactivity', followed by 'prosocial behaviour', 'average problems', and 'conduct problems' for both the parent and teacher ratings.

#### Family status interactions with gender

In addition to the main effects of gender and family status, there were interactions of these two factors for some SDQ scales. The strongest status by gender interaction was present for the 'hyperactivity scale', both for the parent (Q = 40.6) and the teacher ratings (Q = 11.9). This effect was evident in small gender differences for probands but higher differences for siblings: male siblings had scores about twice as high as female siblings (see Table 2 and Table S2 in additional file 6). Similar to this interaction effect, the effect of gender was also more pronounced for siblings than for probands for the scale measuring 'average problems'. These effects were illustrated additionally by overlapping or almost overlapping CI's between girls and boys in 'the probands, but non-overlapping CI's between boys and girls in the siblings. This pattern applied to both parent and teacher ratings and to both 'average problems' and 'hyperactivity'.

#### Main effects of study-centre and its interactions

The effects of study-centre were stronger in all scales for the teacher ratings compared to the parent ratings, except for the scale 'prosocial behaviour' which showed the strongest study-centre effect for the parent ratings (see Q statistics in Table 3). The parents differed across centres also in their ratings of 'conduct problems' and 'hyperactivity', but not of 'emotional problems' and 'peer problems'. The only teacher rating scale with non-significant effects of study-centre was that on 'emotional problems'.

There was a notable centre by status interaction for the parent ratings but not for the teacher ratings. The variation of parental perception of proband-sibling differences across centres was highest for 'hyperactivity' and least pronounced for 'emotional problems' and 'peer problems'.

The parent ratings did not differ with respect to gender effects across centres, and the teacher ratings showed small centre by gender interaction effects only in the scales 'hyperactivity' and 'average problems'.

#### Effects of the informant and the interactions

After controlling for age, status, and gender, the parents provided significantly higher ratings than the teachers on the scales 'conduct problems' (trimmed means 2.9 : 1.7), 'emotional problems' (2.7 : 2.0), and 'average problems' (3.6 : 3.0; see Table 4 Figure 1, and 1 Table S2 in additional file 6). An interaction between family-status and informant was present in all scales except 'peer problems'. This effect resulted from larger proband-sibling differences in parents compared to teachers on the scales for 'conduct problems' (3.0 : 2.0), 'emotional problems' (1.8 : 1.3), and 'hyperactivity' (5.9 : 4.7). The difference between parent and teacher ratings on the scale for 'prosocial behaviour' was only slightly smaller (1.5 : 1.8).

**Table 4:** Strengths and Difficulties Questionnaire (SDQ)

Effects of informant (with gender or status)°								
	***Informant***		***Status × Informant***		***Gender × Informant***		***Status × Gender × Informant***	
				
	***Q***	***p***	***Q***	***p***	***Q***	***p***	***Q***	***p***

***CP***	6.049	0.014	65.184	0.000	2.184	0.140	3.104	0.078
***EP***	12.008	0.001	41.739	0.000	0.745	0.388	2.344	0.126
***H***	2.601	0.107	8.616	0.003	0.308	0.579	0.211	0.646
***PB***	1.946	0.163	44.041	0.000	4.097	0.043	0.982	0.322
***PP***	0.347	0.556	0.308	0.579	2.976	0.085	0.466	0.495
***AvP***	3.897	0.049	31.646	0.000	0.002	0.964	2.336	0.127

**° **Results of robust between (status) by between (gender) by within (informant) ANOVA; dependent variables are adjusted for age; main effects of status and gender, and their interaction, are not shown

***CP ***Conduct Problems

***EP ***Emotional Problems

***H ***Hyperactivity

***PB ***Prosocial Behaviour

***PP ***Peer Problems

***AvP ***Average Problems

***Q ***Q-statistic of robust between × between × within ANOVA

Gender and informant interacted only with the 'prosocial behaviour' scale. This effect was statistically small and resulted from greater parent-teacher differences in boys (1.2) compared to girls (0.8). There were no significant three-way interactions.

### Social communication questionnaire

The SCQ was given only to the parents. The differences in the scores for probands (7.6) and siblings (3.7) and for boys (6.1) and girls (3.5) were quite large and similar in direction. This suggests that there were gender and status effects but no interactions (Table S2 in additional file 6). The three-way statistics showed a large effect of family status (Q = 108), but only a small effect of gender (Q = 6.9), and no status by gender interaction. Centres differed clearly from each other in their mean overall ratings and in the differential perception of probands and siblings, but not in the ratings for boys and girls (Table 3).

### Intelligence

IQ data were available from 842 probands and 1002 siblings. The WAIS-III was applied to 16 probands and 31 siblings who were between 17 and 19 years old. All other children completed the WISC-III.

#### Effects of age

Age was negatively correlated with the prorated IQ (rho = -.106) and the IQ subtests measuring 'vocabulary' (rho = -.14), 'similarities' (rho = -.117), and 'picture completion' (rho = -.060; Table 5). The following analyses were based on age-adjusted IQ measures, i.e. residuals of a linear regression on age.

**Table 5:** Intelligence (Effects of age, status, gender, and Centre)

			***Age effects***				***Centre effects*°**						***Gender effects*°**						***Status effects*°**				
									
	**N**		***rho***		***p***		***Q***		***Crit***		***Sig***		***Q***		***Crit***		***Sig***		***Q***		***Cri*t**		***Sig***
									
***IQ***	1828		-0.106		0.000		91.7		23.4		***		4.49		4.19		*		7.5		4.2		*
***V §***	1844		-0.137		0.000		136.2		21.7		***		8.58		4.01		**		10.9		4.0		**
***S §***	1788		-0.117		0.000		86.9		21.6		***		3.76		4.17				5.1		4.2		*
***PC §***	1788		-0.060		0.012		110.6		21.5		***		8.78		4.03		**		0.7		4.0		
***BD §***	1845		-0.007		0.777		30.5		19.9		**		2.67		3.97				7.7		4.0		**
***DS §***	1820		0.002		0.918		42.7		21.7		***		0.41		4.03				16.3		4.0		***

***IQ ***Prorated IQ

***V ***Vocabulary

***S ***Simliarities

***PC ***Picture Completion

***BD ***Block Design

***DS ***Digit Span

°Three-way analysis; dependent scores are

adjusted for age (residuals of linear regression)

§ Analyses with collapsed israel samples

*Q *Test statistic of robust 3-way effects

Crit α = .05 critical value

Sig Two sided α-level

* p < 0.05

** p < .0.01

*** p < .0.001

#### Effects of family status, gender, and their interaction

The probands had a significantly lower IQ (100.9) than the siblings (102.8), and also lower scores on all subtests except for 'picture completion,' where probands and siblings did not differ significantly, i.e. had overlapping confidence intervals (Figure 1, Table S3 in additional file 7).

Boys had a significantly higher prorated IQ (102.4) than girls (101.1), and higher scores on all subtests except for the 'digit span', where the girls scored higher than the boys. The IQ difference between boys and girls was larger in the probands (3.4) than the siblings (2.1). This difference was also maintained for all subtest scores except for the 'digit span', which did not differ between boys and girls. All other scores were significantly higher in boys than in girls for both probands and siblings.

A statistical multi-way analysis adjusted for age and including study-centre, gender, and status effects was performed. This revealed the effects of family status were stronger (higher Q values) than the effects of gender on the prorated IQ and all subtests except for 'picture completion'. The latter had stronger gender effects than status effects (Table 5). Statistically, the effects of family status, with lower scores for the probands, were significant for all tests except 'picture completion'. In contrast, the effects of gender, with higher scores for boys, were only significant for IQ, 'vocabulary', and 'picture completion.

There were no significant gender by status interactions on any of the IQ measures.

#### Effects of study-centre and the interactions with gender and family status

Subjects from the various centres differed significantly from each other on IQ and all subtest scores. However, there were no interaction effects including centre for any of the subtests and IQ. Twenty post-hoc pairwise comparisons between centres were significant (probability level adjusted for multiple tests; Figure S5 in additional file 8). One centre with a low mean IQ (IRL_D: 94.4) and two centres with high IQ's (SWI_Z: 110.6, and ESP_V: 111.5) contrasted with the other centres that showed continuously distributed IQs from 99.5 to 106.2. Pairwise comparisons between centres for subtest scores were not analysed further as the cell sizes in several centres were too small.

## Discussion

The present paper investigated the influence of age, gender, family status, informant, and recruiting centre on behavioural measures and intelligence in the International Multi-centre ADHD Genetics (IMAGE) project. The issue of homogeneity was of particular interest, because the power necessary for detecting susceptible genes is not only dependent on the sample size, but also on the homogeneity of the sample. Beyond genetic studies, our findings may be of more general interest for ADHD research, at least for study designs comparing clinical indicators of ADHD with other measures, e.g. for the investigation of neuropsychological or neurophysiological markers. Not least, some of our findings are of relevance for clinical practice in ADHD.

We had differing expectations, according to the various categories of data to which our measures can be assigned, about the influence of the different factors on the dependent measures: For example, because the IQ scores were based on language-specific normative samples, we expected effects for gender and family status but not for age and study centre. In the case of the raw-scores of the SDQ and the SCQ scales, we expected effects of age, status, gender, and informant [24,26,47], and probably also effects of study-centre [17]. For the CTRS and CPRS scores, which are based on normative samples reflecting age, gender, and informant, we expected no age and gender and informant effects but status effects and probably centre effects [17].

Our analyses revealed numerous effects of independent factors on behavioural measures and intelligence which were not expected or exceeded the expected range. Many of these effects were present as interactions in addition to or instead of the main effects. To summarize the large number of effects and results, the following discussion focuses particularly on unexpected results or findings that were related to sample heterogeneity. The discussion emphasises the following three main factors that affected the distribution of behavioural measures: 1) The diagnostic procedures 2) the multi-centre design and 3) the source of information.

The formal diagnosis of 'ADHD-CT' for each proband required the presence of both six symptoms of inattention and six symptoms of hyperactivity/impulsivity. The presence of each symptom was given if it was recorded either in the teacher questionnaire or in the parent interview. This diagnostic criterion was not applied to the siblings in terms of an inclusion criterion but, rather, in cases of suspected ADHD as a potential exclusion criterion for the sibling sample in further analyses. Thus, structural differences between proband and sibling samples (effects of family status), such as the gender differences in mean behavioural scores, could reflect differing criteria for inclusion.

A second important issue concerns the effects of pooling behavioural data from different recruiting centres and different countries. A multi-centre design is usually chosen in order to increase the power of statistical inference. We were interested in the amount of heterogeneity, i.e. of additional variance stemming from differences between centres. Heterogeneity could present the other side of the coin with respect to statistical power by decreasing statistical power in subsequent analyses. Informant effects have already been investigated in the first paper [17]. There we showed how diagnostic symptoms were perceived by different informants and instruments. In the present paper we were mainly interested in heterogeneity of the behavioural data stemming from informant effects and interactions with other factors.

As expected, probands had higher scores than siblings on all rating scales from both parent and teacher questionnaires. Similarly, the IQ measures were lower in probands than in siblings, at least in the measures with significant differences. In contrast to the questionnaire measures, the IQ differences had only small effect sizes. In the questionnaires scores with age and gender norms (CTRS and CPRS) status effects interacted with gender effects: female probands deviated to a weaker extent (about one SD less) from the population norm than male probands, particularly on the scales including hyperactive symptoms. In contrast, the differences in the deviation from the normative mean were in the opposite direction in the siblings: male siblings deviated on the relevant ratings by about half an SD more from the normative means than the female siblings.

We interpret this gender by status interaction as a bias which is attributable to the recruitment strategy: The DSM-IV inclusion criterion for ADHD-CT requiring the presence of six inattentive and six hyperactive/impulsive symptoms, independent of gender, led to the higher T-scores in female siblings. Moreover, we found evidence for this recruiting bias also in the hyperactivity scale of the SDQ. This scale is a raw-score and therefore reflects the perceived symptoms without relating them to population distributions. The male siblings had higher scores than the female siblings, reflecting known population differences. In contrast, the scores of male and female probands did not differ from each other, reflecting the symptom based diagnostic strategy.

The stronger deviation from normality in girls with externalizing, particularly hyperactive, symptoms compared to boys with identical symptoms is reflected in the normative samples of the questionnaires [36] and consistent with empirical evidence [24]. Consequently, the male to female ratio in our proband sample was about 7:1 whereas girls and boys were equally frequent in the sibling sample.

Technically, this gender by status interaction effect on questionnaire scores in our sample introduced a gender bias in comparisons between probands and siblings. This bias may not only affect genetic analyses, but also categorical or quantitative analyses of neurobiological or neuropsychological markers. Even in a purely clinical context, one may question the validity of a diagnosis which is based mainly on symptom numbers, independent of epidemiological considerations of gender-specific distributions. In contrast, diagnostic models which would define gender specific liability-thresholds dependent on epidemiological distributions of a trait [14] would lead to almost identical numbers of affected subjects for each gender. It certainly would lead beyond the scope of the present contribution to decide which of the two fundamentally different approaches is of greater benefit for research and for clinical practice. Nevertheless, our finding may contribute to further discussions about the diagnosis of ADHD and future revisions of diagnostic systems.

The effects of family status also interacted with study-centre. In both raw and normative scores, we found centre main effects. These effects were measured either exclusively in the teacher ratings or, on some scales, were higher in the teacher ratings than in the parent ratings.

In contrast, centre by status interaction effects were present only in the parent ratings. These interactions were expressed in the greater number of pairwise centre differences, e.g. in ADHD DSM-IV scores of the Conners' questionnaires, in probands than in siblings. To put it the other way around: proband - sibling differences varied markedly across sites (e.g. about 0.8 SD for the centre ISR_P, but about 2.7 SD for IRL_D). It is not possible to provide a clear explanation for this phenomenon. Because we also found similar effects in the raw scores of the SDQ, we may perhaps exclude the use of a single (US) normative sample as a confounding factor of influence in the Conners' questionnaires. Furthermore, sociocultural normative backgrounds attributable to countries can explain only a part of the variance, because gender differences did not cluster in national categories.

Furthermore, status effects also interacted with informant effects, independent of the influence of centres. In contrast, there were no main effects of informants in the hyperactivity scores. The status by informant interaction was evident mainly in larger proband-sibling differences in the parent ratings compared to the teacher ratings. These interactions were considerable in raw and in normative scores and mostly concerned ADHD symptom scales or social behaviour ratings. In general, the siblings were perceived similarly by parents and teachers, both in raw scores and normative scores. In contrast, the probands had higher scores in the parent than in the teacher ratings. We conclude that the contrast effects [48] were more due to symptom aggravation in the parents perception of the probands behaviour than to suppression of their perception in the behaviour of the siblings. Again, this interaction between informant and family status resulting in higher contrasts in the parent ratings than in the teacher ratings introduces further heterogeneity to the sample. If not taken into account, this interaction may reduce statistical power in statistical analyses, even if average scores are used.

Effects of the study centre were discussed already in the context of their interaction with family status. A statistical main effect of centre was present mainly in the teacher ratings and weak to absent in the parent ratings. Because statistically testing of the interaction between centres and informant, for reasons of the data structure, was not possible, this differential main effect can be interpreted as a centre by informant interaction, even without statistical evidence. A definite interpretation of this effect is difficult. National or centre specific factors may have played a role. However, a simple pattern was not recognisable, because significant differences between centres were not consistent across the variables analysed (These were the DSM-IV ADHD scores and the oppositional score of the Conners' questionnaire).

In contrast to these rather weak effects, IQ differed to a greater extent between centres. Unlike the questionnaire scores, IQ data were collected by trained clinicians. The remarkable mean differences across centres (e.g. 17 IQ points difference between IRL_D and ESP_V) do not seem to reflect sociocultural differences between regions or countries, because the use of language specific normative samples should have accounted for them. The greatest difference between the three German speaking centres (GER_G, GER_E, and SWI_Z) all using the same normative sample was 8.5 IQ points. We speculate that differences in sampling strategies (existing patient register, outpatient or inpatient clinic, self-help organisations, resident doctors, newspaper advertisements etc.) may have played a role. Additionally, different test settings may have influenced the results: some assessments were included in a neuropsychological test battery, others were not, and in some cases pre-existing recent IQ assessments were used.

Finally, informant effects were present in various forms. Significant informant effects were recorded mainly in scales to which ADHD symptoms contributed at most only marginally, namely, in two scales of the SDQ (Conduct Problems and Emotional Problems) and in two normative scales of the Conners' questionnaires (Cognitive Problems and Social Problems). In contrast, informant effects were absent in the Hyperactivity scale of the SDQ and in the ADHD scales of the Conners' questionnaires. Although the ADHD scores did not differ between the raters in terms of an informant main effect, they were differently influenced by the raters depending on the family status. This informant by status interaction (contrast effects) has already been discussed above.

Compared to the informant by status interaction effects, the informant by gender interactions were weaker and, in combination with three way informant by status by gender effects, are more difficult to interpret. Mean score comparisons indicated larger differences between the parent and teacher ratings in boys, but not in girls. But these differences should be interpreted cautiously because there were major differences in the male to female ratios among the probands but not among the siblings. In addition, it should be noted that significant effects were found mainly in the normative scales. Thus, the reported differences did not necessarily reflect differences in the perceived behaviour but rather in the deviation from the normative mean. Given the rather small effects and the complexity of interacting factors we refrain from further interpretation of gender interactions.

In summary, first we found remarkable main effects of the study centre and interactions of centres with questionnaire scores and IQ even though a standardised recruiting procedure was employed. We assume that an interplay between local and national factors, between recruiting strategies and sociocultural aspects may explain these effects. Our data provide evidence for at least questioning to some extent the benefit of multi-centre designs. The statistical power achieved by enlarging the sample size may be lost by the additional heterogeneity introduced by the use of different centres.

Secondly, our data provide evidence for a remarkable heterogeneity in the behavioural data as a result of the use of symptom based diagnostic criteria, which reflect the actual state of the art. Boys and girls differed from normality to a considerably different extent despite the similar profile of their symptoms. In addition, the probands and siblings differed on several features that could be attributable to the diagnostic procedure, such as the gender differences shown on the questionnaire ratings.

## Conclusion

We conclude that multi-centre studies not only offer better conditions for statistical analyses by the increase in sample size, but may also increase the heterogeneity in the behavioural data counteracting the gain of statistical power gained by the larger sample size. Additionally, we question the present state of the art in ADHD diagnosis leading to inadvertent distortions of the sample in terms of deviations from normality and in many cases also in terms of the underlying genotype. This heterogeneity may reduce the power in statistical analyses investigating associations between behavioural data and their correlates at a neuronal or genetic level.

## Competing interests

PA has consulted with, received education grants from or spoken at sponsored meetings for Shire, Janssen-Cilag, Eli-Lilly and Flynn Pharma. JB has been in the past 3 years a consultant to/member of advisory board of/and/or speaker for Janssen Cilag BV, Eli Lilly, Bristol-Myer Squibb, Organon/Shering Plough, UCB, Shire, Medice, Servier, and Servier. TB served in an advisory or consultancy role for Bristol-Myers Squibb, Desitin, Lilly, Medice, Novartis, Pfizer, Shire, UCB and Viforpharma. He received conference attendance support or received speaker's fee by Lilly, Janssen McNeil, Medice, Novartis, Shire, UCB. He received unrestricted grants for organizing a CME conference by Lilly, Janssen McNeil, Medice, Novartis, Shire, UCB. He is/has been involved in clinical trials conducted by Lilly, Shire and Novartis. The present work is unrelated to the above grants and relationships. SVF has, in the past year received consulting fees and has been on Advisory Boards for Eli Lilly, Ortho-McNeil and Shire Development and has received research support from Eli Lilly, Pfizer, Shire and the National Institutes of Health. In previous years, SVF has received consulting fees or has been on Advisory Boards or has been a speaker for the following sources: Shire, McNeil, Janssen, Novartis, Pfizer and Eli Lilly. In previous years he has received research support from Eli Lilly, Shire, Pfizer and the National Institutes of Health. RDO received support from Janssen and UCB during this period. HR has served as an advisor to Shire and received research support from Shire and Lilly and conference attendance support from Lilly. The present study is unrelated to these relationships. AR declares the following competing interests: Advisory Board and Speakers Bureau: Lilly, Shire, Medice, Novartis; Research Support: Shire, German Research Society, Schwaabe; Travel Support: Shire; Educational Grant: Shire. JS declares the following competing interests: Advisory Board: Lilly, Shire, Research Grant(s) Lilly, Speaker's Fee: Shire, Lilly, Janssen-Cillag. ESB declares the following competing interests: Recent speaker board: Shire, UCB Pharma. Current & recent consultancy: UCB Pharma, Shire. Current & recent research support: Janssen Cilag, Shire, Qbtech, Flynn Pharma. Advisory Board: Shire, Flynn Pharma, UCB Pharma, Astra Zeneca. Conference support: Shire. HCS has served as an advisor and/or speaker to the following companies: Janssen-Cilag, Eli Lilly, Medice, Novartis, Shire, and UCB. MT has served as an advisor, speaker and had research grants from the following companies: Janssen-Cilag, Eli Lilly, Shire, and UCB. All other authors declare no competing interests to disclose.

## Authors' contributions

UCM and HCS jointly planned the analyses and drafted the manuscript with UCM performing all the statistical analyses. All other authors were principle investigators at the various centres and SVF was overall principle investigator of the IMAGE study. All authors commented on the manuscript and approved the final draft.

## Pre-publication history

The pre-publication history for this paper can be accessed here:

http://www.biomedcentral.com/1471-244X/11/55/prepub

## Supplementary Material

Additional file 1 **Table S1**. Quantiles and trimmed means with confidence intervals of Conners' Questionnaires. Click here for file

Additional file 2 **Figure S1**. Histograms of Conners' rating scales (CTRS-R:L, CPRS-R:L). Click here for file

Additional file 3 **Figure S2**. Post-hoc comparisons of selected Conners' Parent Rating Scales (A, L, M, N). Click here for file

Additional file 4 **Figure S3**. Post-hoc comparisons of selected Conners' Teacher Rating Scales (A, L, M, N). Click here for file

Additional file 5 **Figure S4**. Histograms of the Strengths and Difficulties Questionnaire (SDQ) and the Social Communications Questionnaire (SCQ). Click here for file

Additional file 6 **Table S2**. Quantiles and trimmed means with confidence intervals of the Strengths and Difficulties Questionnaire (SDQ) and the Social Communications Questionnaire (SCQ). Click here for file

Additional file 7 **Table S3**. Quantiles and trimmed means with confidence intervals of intelligence measures. Click here for file

Additional file 8 **Figure S5**. Post-hoc comparisons of the prorated IQ. Click here for file
