# Supplementary material for: The impact of study design and diagnostic approach in a large multi-centre ADHD study: Part 2: Dimensional measures of psychopathology and intelligence
Source: BMC Psychiatry. 2011 Apr 7;11:55. doi: 10.1186/1471-244X-11-55 (PMC3090338; doi:10.1186/1471-244X-11-55)
Supplement: Additional file 1 — Table S1. Quantiles and trimmed means with confidence intervals of Conners' Questionnaires. [file 1471-244X-11-55-S1.PDF]

Table S1: Conners' Questionnaires

Parent ratings

	Male Probands (n=938)						Male Siblings (n=730)						All boys (n=1668)					
	Q25	Q50	Q75	$mean_t$	$Ci_{t,low}$	$Ci_{t,up}$	Q25	Q50	Q75	$mean_t$	$Ci_{t,low}$	$Ci_{t,up}$	Q25	Q50	Q75	$mean_t$	$Ci_{t,low}$	$Ci_{t,up}$
A	62	72	80	**71.1**	70.2	72.0	46	54	66	**54.9**	53.7	56.1	52	65	76	**64.7**	63.8	65.7
B	64	71	76	**70.2**	69.6	70.8	45	54	65	**54.1**	53.0	55.3	54	66	73	**64.8**	64.1	65.6
C	74	81	90	**81.9**	81.1	82.7	46	53	70	**55.2**	53.9	56.6	56	74	87	**72.6**	71.4	73.7
D	47	55	68	**56.6**	55.6	57.6	45	50	58	**50.7**	49.9	51.6	46	53	65	**53.9**	53.2	54.6
E	46	55	63	**54.6**	53.7	55.6	43	48	57	**48.8**	48.0	49.7	44	52	60	**52.0**	51.4	52.6
F	53	65	80	**66.0**	64.7	67.3	45	49	60	**50.4**	49.5	51.3	49	57	73	**58.9**	57.9	59.9
G	47	58	69	**56.9**	55.7	58.1	43	48	58	**49.6**	48.6	50.7	43	53	67	**53.5**	52.7	54.4
H	68	74	78	**73.4**	72.9	73.9	45	54	67	**54.7**	53.5	55.9	56	69	76	**67.5**	66.7	68.3
I	71	77	84	**77.6**	76.9	78.2	45	54	68	**55.5**	54.2	56.8	56	72	81	**70.3**	69.3	71.2
J	57	67	79	**67.6**	66.5	68.7	43	52	65	**53.4**	52.2	54.6	51	61	73	**61.7**	60.7	62.6
K	70	76	83	**76.5**	75.8	77.2	45	54	69	**55.5**	54.2	56.8	56	70	80	**69.4**	68.5	70.3
L	66	71	76	**71.3**	70.7	71.9	44	53	66	**53.6**	52.5	54.8	54	67	74	**65.6**	64.8	66.3
M	72	80	88	**80.2**	79.5	81.0	45	53	68	**54.6**	53.3	56.1	56	74	84	**71.3**	70.2	72.4
N	71	77	83	**77.1**	76.4	77.7	45	54	67	**55.0**	53.8	56.3	56	71	80	**69.9**	68.9	70.8
Mean	62	70	78	**70.1**	69.2	70.9	45	52	65	**53.3**	52.2	54.5	52	65	75	**64.0**	63.1	64.9

	Female Probands (n=130)						Female Siblings (n=716)						All girls (n=846)					
	Q25	Q50	Q75	$mean_t$	$Ci_{t,low}$	$Ci_{t,up}$	Q25	Q50	Q75	$mean_t$	$Ci_{t,low}$	$Ci_{t,up}$	Q25	Q50	Q75	$mean_t$	$Ci_{t,low}$	$Ci_{t,up}$
A	62	75	83	**73.6**	70.8	76.3	44	50	60	**51.3**	50.4	52.3	45	53	66	**54.2**	52.9	55.3
B	72	82	88	**81.0**	78.8	83.2	44	49	61	**51.2**	50.2	52.2	45	54	70	**55.4**	53.9	56.8
C	83	90	90	**88.4**	86.9	89.3	44	49	61	**50.4**	49.5	51.6	44	52	75	**55.4**	53.8	57.2
D	49	62	75	**62.0**	58.7	65.7	42	49	59	**49.6**	48.7	50.5	43	50	61	**51.0**	50.0	52.1
E	46	53	64	**53.4**	50.9	55.9	42	47	54	**47.4**	46.8	48.1	43	48	54	**48.1**	47.5	48.8
F	59	74	88	**73.1**	69.1	76.9	45	50	56	**48.8**	48.1	49.6	45	50	62	**51.1**	50.2	52.2
G	51	62	80	**63.6**	59.5	67.5	43	47	60	**50.2**	49.2	51.3	43	51	63	**51.8**	50.7	52.9
H	75	84	90	**83.7**	81.6	85.5	44	49	62	**50.5**	49.4	51.7	44	52	72	**55.1**	53.6	56.7
I	78	90	90	**86.4**	84.4	88.1	43	50	62	**51.1**	50.0	52.2	45	53	72	**55.7**	54.2	57.4
J	60	72	82	**70.7**	67.5	74.2	42	48	60	**49.8**	48.9	50.9	43	52	65	**52.5**	51.3	53.8
K	74	85	90	**84.3**	82.1	86.3	44	49	62	**51.3**	50.2	52.4	45	53	72	**55.7**	54.1	57.1
L	74	84	90	**82.7**	80.5	84.7	43	49	62	**50.6**	49.6	51.7	44	53	71	**55.1**	53.6	56.5
M	80	89	90	**86.9**	85.2	88.3	44	49	60	**50.8**	49.9	51.8	46	51	73	**55.4**	53.9	57.1
N	80	90	90	**87.1**	85.5	88.3	44	49	63	**51.1**	50.1	52.3	45	52	75	**56.1**	54.5	57.7
Mean	67	78	85	**76.9**	74.4	79.3	43	49	60	**50.3**	49.4	51.3	44	52	68	**53.8**	52.5	55.1

Teacher ratings

	Male Probands (n=938)						Male Siblings (n=730)						All boys (n=1668)						
	Q25	Q50	Q75	$mean_t$	$Ci_{t,low}$	$Ci_{t,up}$	Q25	Q50	Q75	$mean_t$	$Ci_{t,low}$	$Ci_{t,up}$	Q25	Q50	Q75	$mean_t$	$Ci_{t,low}$	$Ci_{t,up}$	
A	55	65	76	**65.2**	64.1	66.4	45	51	63	**52.1**	51.1	53.2	48	58	72	**59.6**	58.7	60.5	***A***
B	55	62	68	**61.7**	61.0	62.4	46	52	61	**52.6**	51.7	53.6	50	59	66	**58.3**	57.7	58.9	***B***
C	62	70	78	**70.2**	69.5	71.0	46	53	64	**53.6**	52.6	54.6	53	64	74	**63.9**	63.1	64.8	***C***
D	55	63	73	**64.0**	63.1	64.9	51	57	67	**57.8**	56.8	58.8	53	61	71	**61.3**	60.6	62.0	***D***
E	49	55	64	**55.6**	54.8	56.4	46	50	58	**51.1**	50.3	52.0	46	53	62	**53.6**	53.0	54.2	***E***
F	48	58	69	**58.1**	57.0	59.1	45	48	56	**49.0**	48.3	49.7	45	52	64	**53.7**	53.0	54.5	***F***
G																			***G***
H	65	71	76	**70.7**	70.1	71.3	46	55	66	**55.3**	54.2	56.4	55	66	74	**65.3**	64.6	66.1	***H***
I	64	72	78	**71.7**	71.0	72.3	48	55	67	**55.9**	54.8	57.1	55	67	75	**66.0**	65.3	66.8	***I***
J	57	69	81	**68.6**	67.4	69.9	45	51	63	**52.7**	51.6	53.9	49	62	74	**61.7**	60.7	62.8	***J***
K	66	73	80	**73.0**	72.2	73.8	47	56	66	**56.1**	54.9	57.2	55	67	76	**66.6**	65.8	67.4	***K***
L	61	66	72	**66.2**	65.6	66.8	46	54	64	**54.1**	53.1	55.2	53	62	70	**62.0**	61.3	62.6	***L***
M	61	69	76	**69.3**	68.5	70.1	44	51	62	**52.2**	51.2	53.2	51	63	73	**62.7**	61.8	63.5	***M***
N	64	69	75	**69.1**	68.5	69.7	46	53	64	**54.0**	53.0	55.0	54	65	72	**63.8**	63.1	64.5	***N***
Mean	59	66	74	**66.4**	65.6	67.2	46	53	63	**53.6**	52.6	54.6	51	61	71	**61.4**	60.7	62.2	*Mean*

	Female Probands (n=130)						Female Siblings (n=716)						All girls (n=846)						
	Q25	Q50	Q75	$mean_t$	$Ci_{t,low}$	$Ci_{t,up}$	Q25	Q50	Q75	$mean_t$	$Ci_{t,low}$	$Ci_{t,up}$	Q25	Q50	Q75	$mean_t$	$Ci_{t,low}$	$Ci_{t,up}$	
A	55	65	86	**66.5**	62.6	70.5	46	46	60	**49.7**	48.9	50.6	46	51	64	**51.7**	50.8	52.9	***A***
B	57	66	78	**66.6**	63.9	69.5	45	51	62	**52.3**	51.3	53.3	46	54	64	**54.5**	53.4	55.5	***B***
C	70	85	90	**82.3**	79.1	85.0	46	50	62	**52.5**	51.4	53.6	44	46	54	**56.3**	54.8	57.9	***C***
D	55	66	79	**66.0**	62.8	69.2	50	55	66	**57.0**	56.0	58.1	50	58	67	**58.3**	57.3	59.3	***D***
E	48	53	59	**52.7**	51.0	54.4	45	50	58	**50.7**	49.9	51.5	45	50	58	**51.0**	50.3	51.7	***E***
F	50	61	76	**61.6**	58.0	65.6	46	46	56	**48.5**	47.8	49.4	46	46	61	**50.1**	49.2	51.0	***F***
G																			***G***
H	71	82	90	**82.2**	79.6	84.5	45	51	66	**53.3**	52.1	54.5	46	55	74	**57.4**	55.9	59.0	***H***
I	73	87	90	**82.9**	80.5	85.1	44	51	65	**53.5**	52.3	54.9	47	55	74	**57.8**	56.3	59.5	***I***
J	53	68	90	**70.9**	66.4	75.2	45	46	61	**50.0**	49.0	51.1	45	51	68	**52.4**	51.2	53.7	***J***
K	71	85	90	**82.3**	79.5	84.7	46	52	65	**53.3**	52.1	54.7	47	55	75	**57.6**	56.1	59.2	***K***
L	65	73	83	**73.9**	71.5	76.4	45	53	62	**53.1**	52.0	54.3	46	55	69	**56.3**	55.1	57.6	***L***
M	69	85	90	**81.8**	78.4	84.5	45	48	61	**50.7**	49.7	51.8	42	45	51	**54.5**	53.1	56.1	***M***
N	71	81	90	**80.4**	77.9	82.6	46	52	64	**53.3**	52.2	54.5	46	56	72	**57.2**	55.8	58.7	***N***
Mean	62	74	84	**73.1**	70.1	75.9	46	50	62	**52.1**	51.1	53.3	46	52	65	**55.0**	53.8	56.3	*Mean*

Table A1: Conners' Questionnaires (continued)

Parent ratings

	All Probands (n=1068)						All Siblings (n=1446)						All (n=2514)					
	Q25	*Q50*	*Q75*	*mean $_t$*	*Ci $_{t,low}$*	*Ci $_{t,up}$*	*Q25*	*Q50*	*Q75*	*mean $_t$*	*Ci $_{t,low}$*	*Ci $_{t,up}$*	*Q25*	*Q50*	*Q75*	*mean $_t$*	*Ci $_{t,low}$*	*Ci $_{t,up}$*
A	62	72	80	**71.4**	70.5	72.3	45	52	63	**53.0**	52.3	53.8	49	61	74	**61.2**	61.2	61.2
B	65	72	77	**71.1**	70.5	71.6	45	52	63	**52.7**	51.9	53.5	49	63	73	**61.9**	61.9	61.9
C	74	83	90	**82.8**	82.0	83.5	44	51	65	**52.8**	51.9	53.7	49	68	85	**67.0**	66.0	68.1
D	47	55	68	**57.2**	56.2	58.2	44	50	58	**50.3**	49.6	50.9	45	53	63	**53.0**	52.4	53.6
E	46	55	63	**54.5**	53.6	55.4	43	48	55	**48.1**	47.5	48.6	44	50	59	**50.6**	50.1	51.0
F	53	65	81	**66.8**	65.5	68.0	45	49	59	**49.6**	49.0	50.2	45	55	69	**56.1**	55.4	56.9
G	47	58	71	**57.6**	56.4	58.8	43	48	60	**49.9**	49.2	50.6	43	53	65	**52.9**	52.2	53.6
H	69	75	79	**74.2**	73.6	74.6	44	51	65	**52.6**	51.7	53.4	49	65	76	**63.7**	62.9	64.4
I	71	79	85	**78.4**	77.8	79.0	45	52	65	**53.2**	52.3	54.1	50	67	79	**65.7**	64.8	66.6
J	57	67	79	**68.0**	66.9	69.0	43	51	62	**51.6**	50.7	52.4	47	59	72	**58.7**	58.0	59.4
K	70	77	85	**77.3**	76.5	78.0	45	52	66	**53.4**	52.5	54.2	49	67	79	**65.1**	64.2	65.9
L	67	73	78	**72.2**	71.6	72.7	44	51	64	**52.1**	51.3	53.0	49	64	74	**62.3**	61.6	63.1
M	74	81	89	**81.1**	80.3	81.8	44	51	65	**52.7**	51.8	53.5	49	68	82	**66.1**	65.1	67.1
N	71	78	85	**78.1**	77.4	78.7	44	51	65	**53.0**	52.2	53.9	49	67	79	**65.5**	64.6	66.5
Mean	62	71	79	**70.8**	69.9	71.5	44	51	63	**51.8**	51.0	52.6	48	61	74	**60.7**	60.0	61.4

Teacher ratings

	All Probands (n=1068)						All Siblings (n=1446)						All (n=2514)						
	Q25	*Q50*	*Q75*	*mean $_t$*	*Ci $_{t,low}$*	*Ci $_{t,up}$*	*Q25*	*Q50*	*Q75*	*mean $_t$*	*Ci $_{t,low}$*	*Ci $_{t,up}$*	*Q25*	*Q50*	*Q75*	*mean $_t$*	*Ci $_{t,low}$*	*Ci $_{t,up}$*	
A	55	65	76	**65.3**	64.2	66.4	46	49	60	**51.0**	50.3	51.7	46	55	70	**56.9**	56.2	57.7	***A***
B	55	62	69	**62.1**	61.5	62.8	45	52	62	**52.5**	51.8	53.2	49	57	66	**57.2**	56.6	57.7	***B***
C	64	71	80	**71.1**	70.3	71.9	46	51	62	**53.1**	52.4	53.8	50	62	74	**61.6**	60.8	62.3	***C***
D	55	63	74	**64.1**	63.3	65.0	50	57	67	**57.4**	56.7	58.1	51	60	70	**60.3**	59.7	60.9	***D***
E	49	55	64	**55.2**	54.5	56.0	45	50	58	**50.9**	50.4	51.5	46	52	60	**52.7**	52.3	53.2	***E***
F	48	59	70	**58.4**	57.4	59.5	46	46	56	**48.9**	48.4	49.4	46	50	64	**52.6**	52.0	53.2	***F***
G																			***G***
H	65	71	78	**71.6**	71.0	72.2	46	53	66	**54.3**	53.5	55.2	50	64	74	**62.9**	62.3	63.7	***H***
I	65	73	80	**72.5**	71.9	73.2	45	54	66	**54.8**	53.9	55.7	51	64	75	**63.5**	62.8	64.3	***I***
J	57	68	81	**68.8**	67.6	70.0	45	51	63	**51.3**	50.6	52.1	45	57	74	**58.5**	57.6	59.3	***J***
K	66	73	82	**73.8**	73.1	74.6	47	54	66	**54.7**	53.8	55.6	51	64	76	**63.8**	63.1	64.6	***K***
L	61	67	73	**66.8**	66.3	67.4	46	53	64	**53.6**	52.9	54.4	50	61	70	**60.3**	59.7	60.9	***L***
M	62	71	79	**70.2**	69.4	71.1	45	49	62	**51.5**	50.8	52.2	48	60	73	**60.1**	59.3	60.9	***M***
N	64	70	77	**70.0**	69.4	70.7	46	53	64	**53.7**	52.9	54.4	50	62	72	**61.7**	61.1	62.4	***N***
Mean	59	67	76	**66.9**	66.1	67.7	46	52	63	**52.9**	52.2	53.6	49	59	71	**59.4**	58.7	60.1	*Mean*

A Oppositional
B Cognitive Problems / Inattention
C Hyperactivity
D Anxious-Shy
E Perfectionism
F Social Problems
G Psychosomatic
H Conners' ADHD-Index
I Conners' Global Index: Restless-Impulsive
J Conners' Global Index: Emotional Lability
K Conners' Global Index: Total
L DSM-IV: Inattentive
M DSM-IV: Hyperactive-Impulsive
N DSM-IV: Total

Q25 25th quantile
Q50 Median
Q75 75th quantile
mean $_t$ 20% trimmed mean
Ci $_{t, low}$ 95% Confidence interval for trimmed mean (lower end)
Ci $_{t, up}$ 95% Confidence interval for trimmed mean (upper end)
