# Supplementary figures and images for: The impact of study design and diagnostic approach in a large multi-centre ADHD study: Part 2: Dimensional measures of psychopathology and intelligence
Source: BMC Psychiatry. 2011 Apr 7;11:55. doi: 10.1186/1471-244X-11-55 (PMC3090338; doi:10.1186/1471-244X-11-55)

**Figure S1. Histograms of Conners' Rating Scales (CTRS-R:L, CPRS-R:L)**

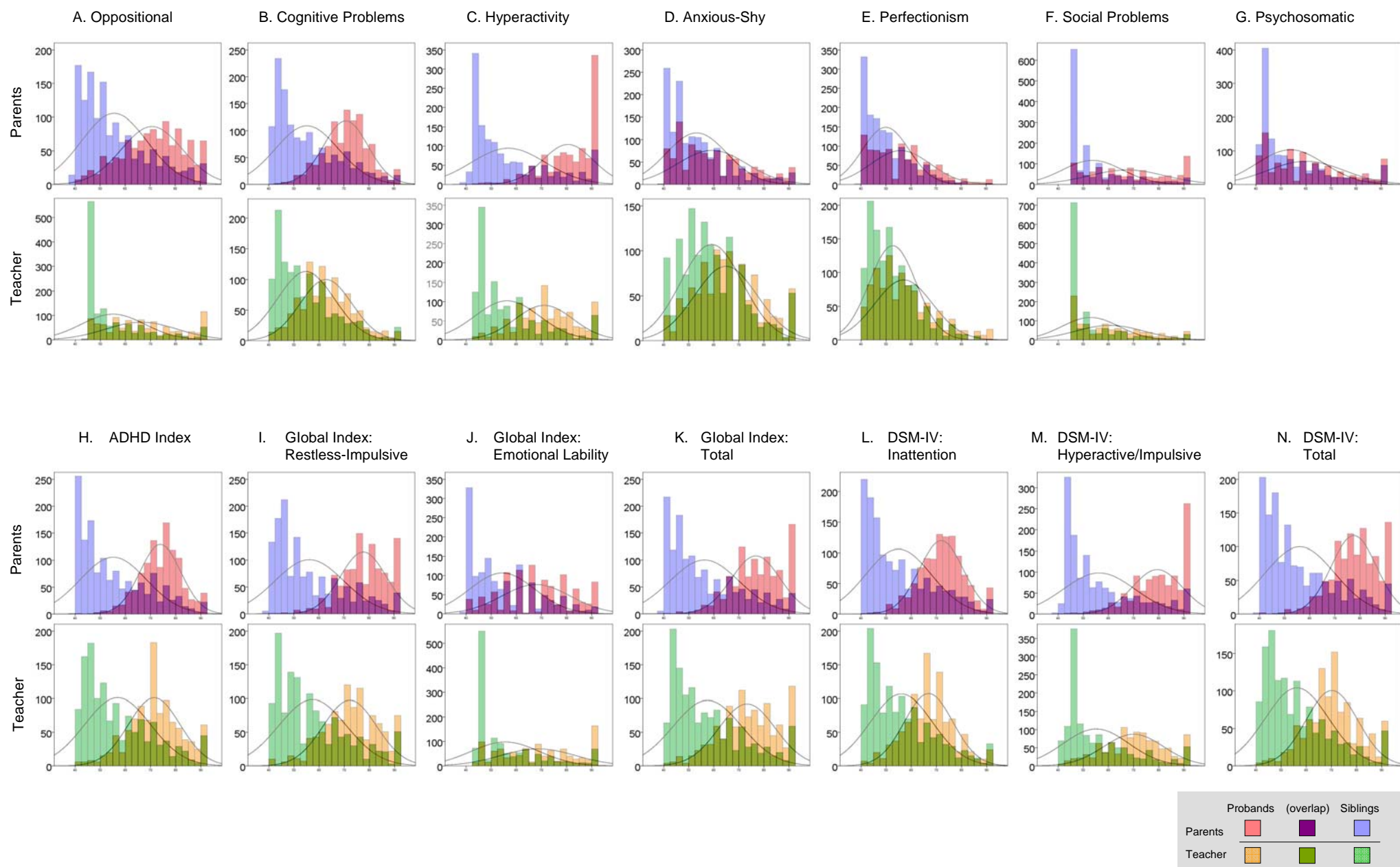

Supplement: Additional file 2 — Figure S1. Histograms of Conners' rating scales (CTRS-R:L, CPRS-R:L). [file 1471-244X-11-55-S2.PDF]
