# Supplementary material for: The impact of study design and diagnostic approach in a large multi-centre ADHD study: Part 2: Dimensional measures of psychopathology and intelligence
Source: BMC Psychiatry. 2011 Apr 7;11:55. doi: 10.1186/1471-244X-11-55 (PMC3090338; doi:10.1186/1471-244X-11-55)
Supplement: Additional file 3 — Figure S2. Post-hoc comparisons of selected Conners' Parent Rating Scales (A, L, M, N). [file 1471-244X-11-55-S3.PDF]

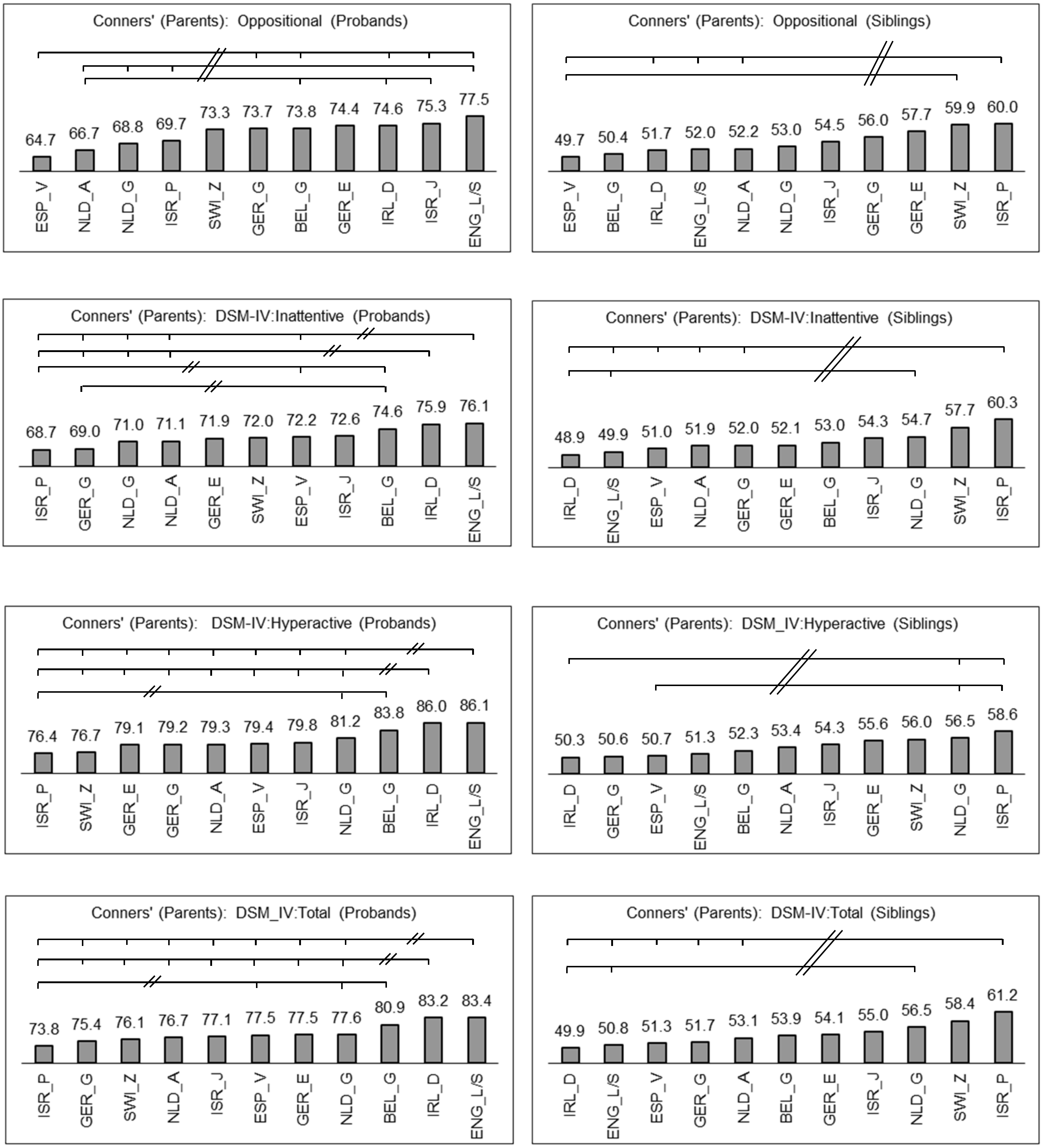


**Figure S2**

**Trimmed means and significant pairwise post-hoc differences of selected Conners' parent scores in probands (n=1068) and siblings (n=1446).**

Notes: Figures show trimmed means per site in ascending order. Horizontal lines above the bars indicate subgroups of significant pairwise differences. Each site indicated by a small vertical line at the left side of the double slash (//) significantly differs from each site indicated by a vertical line on the right side of the double slash, defined as non-overlapping 95% familywise confidence intervals. For centre abbreviations see text.
