# Supplementary material for: The impact of study design and diagnostic approach in a large multi-centre ADHD study: Part 2: Dimensional measures of psychopathology and intelligence
Source: BMC Psychiatry. 2011 Apr 7;11:55. doi: 10.1186/1471-244X-11-55 (PMC3090338; doi:10.1186/1471-244X-11-55)
Supplement: Additional file 4 — Figure S3. Post-hoc comparisons of selected Conners' Teacher Rating Scales (A, L, M, N). [file 1471-244X-11-55-S4.PDF]

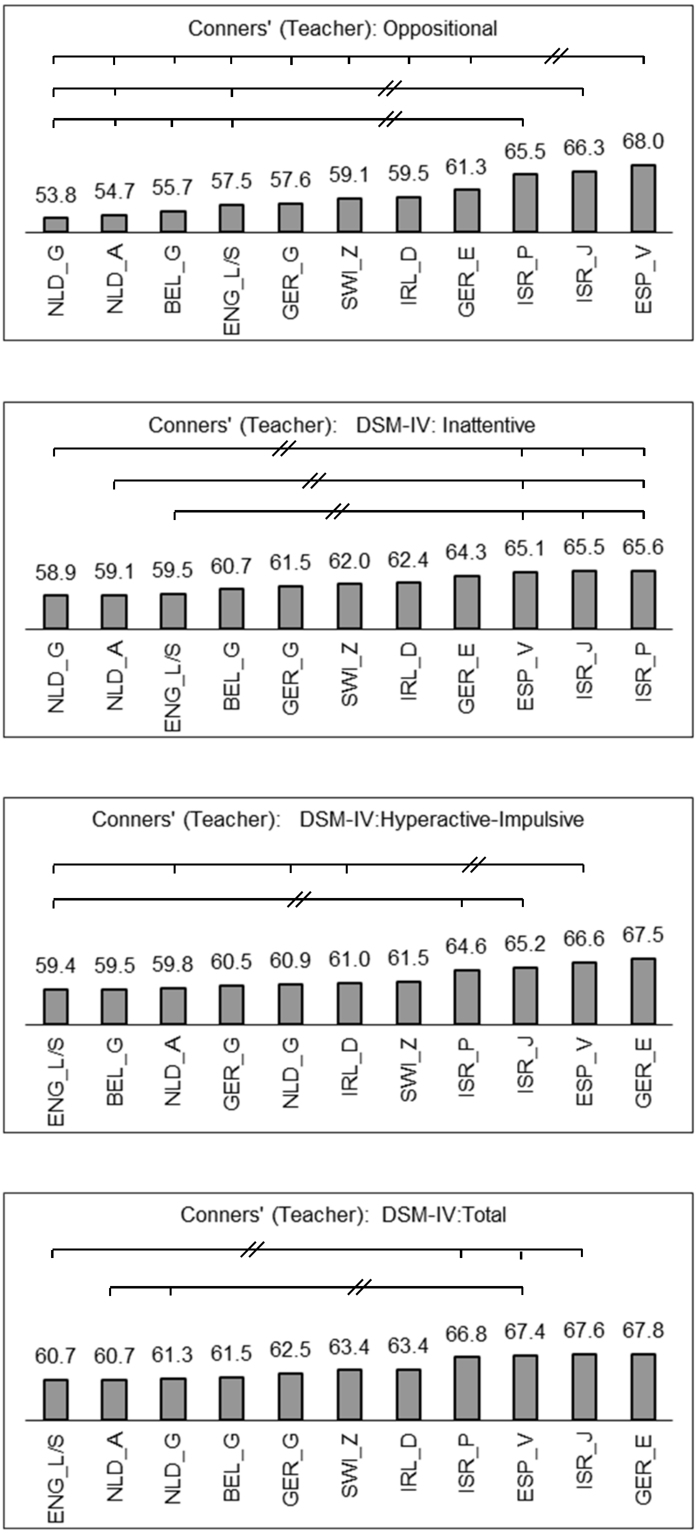

Figure S3

Trimmed means and significant pairwise differences of selected Conners' Teacher scores (n=2514).

Notes: Figures show trimmed means per Centre in ascending order. Horizontal lines above the bars indicate subgroups of significant pairwise differences. Each Centre indicated by the small vertical lines at the left side of the double slash (//) significantly differs from each Centre indicated by vertical lines on the right side of the double slash, defined as non-overlapping 95% familywise confidence intervals. For Centre abbreviations see text.
