# Supplementary material for: The impact of study design and diagnostic approach in a large multi-centre ADHD study: Part 2: Dimensional measures of psychopathology and intelligence
Source: BMC Psychiatry. 2011 Apr 7;11:55. doi: 10.1186/1471-244X-11-55 (PMC3090338; doi:10.1186/1471-244X-11-55)

Figure S4. Strengths and Difficulties Questionnaire (SDQ) / Social Communication Questionnaire (SCQ)

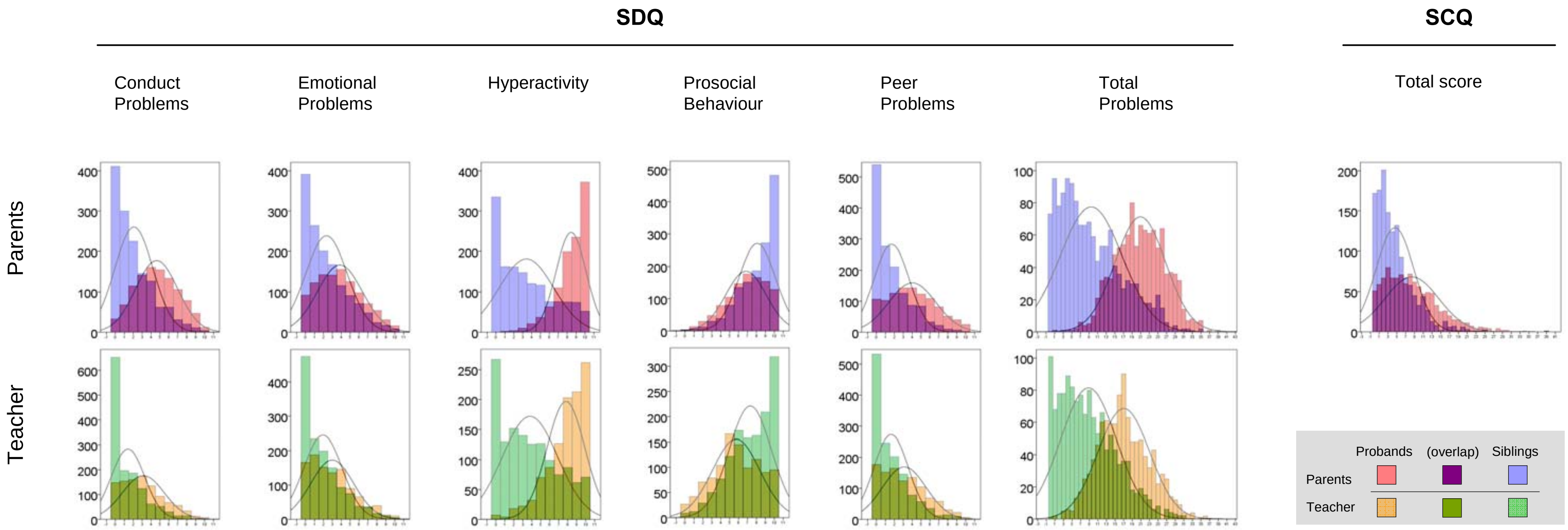

Supplement: Additional file 5 — Figure S4. Histograms of the Strengths and Difficulties Questionnaire (SDQ) and the Social Communications Questionnaire (SCQ). [file 1471-244X-11-55-S5.PDF]
