# Supplementary material for: The impact of study design and diagnostic approach in a large multi-centre ADHD study: Part 2: Dimensional measures of psychopathology and intelligence
Source: BMC Psychiatry. 2011 Apr 7;11:55. doi: 10.1186/1471-244X-11-55 (PMC3090338; doi:10.1186/1471-244X-11-55)
Supplement: Additional file 6 — Table S2. Quantiles and trimmed means with confidence intervals of the Strengths and Difficulties Questionnaire (SDQ) and the Social Communications Questionnaire (SCQ). [file 1471-244X-11-55-S6.PDF]

Table S2: Strengths and Difficulties Questionnaire (SDQ) and Social Communication Questionnaire (SCQ)

Parent ratings

	Male Probands (n=938)						Male Siblings (n=730)						All boys (n=1668)					
	Q25	*Q50*	*Q75*	*mean $_t$*	*Ci $_{t,low}$*	*Ci $_{t,up}$*	*Q25*	*Q50*	*Q75*	*mean $_t$*	*Ci $_{t,low}$*	*Ci $_{t,up}$*	*Q25*	*Q50*	*Q75*	*mean $_t$*	*Ci $_{t,low}$*	*Ci $_{t,up}$*
CP	3	5	6	**4.6**	4.4	4.8	1	2	4	**2.1**	1.9	2.3	2	4	6	**3.5**	3.4	3.7
EP	2	4	5	**3.6**	3.4	3.8	0	0	2	**1.9**	1.7	2.1	1	3	5	**2.9**	2.7	3.0
H	8	9	10	**8.8**	8.7	9.0	1	4	7	**3.9**	3.6	4.2	4	8	9	**7.2**	7.0	7.4
PB(i)	1	3	5	**3.0**	2.9	3.2	1	2	4	**2.1**	1.9	2.3	1	3	4	**2.6**	2.5	2.8
PP	2	4	6	**3.9**	3.7	4.1	0	1	3	**1.6**	1.4	1.8	1	3	5	**2.8**	2.7	3.0
AvP	4	5	6	**5.2**	5.1	5.3	1	3	4	**2.6**	2.4	2.7	3	4	6	**4.3**	4.1	4.4
SCQ	3	8	12	**7.6**	7.2	8.0	2	4	8	**4.5**	4.2	4.8	3	6	10	**6.1**	5.9	6.4

	Female Probands (n=130)						Female Siblings (n=716)						All girls (n=846)					
	Q25	*Q50*	*Q75*	*mean $_t$*	*Ci $_{t,low}$*	*Ci $_{t,up}$*	*Q25*	*Q50*	*Q75*	*mean $_t$*	*Ci $_{t,low}$*	*Ci $_{t,up}$*	*Q25*	*Q50*	*Q75*	*mean $_t$*	*Ci $_{t,low}$*	*Ci $_{t,up}$*
CP	3	4	6	**4.4**	3.9	5.1	0	1	2	**1.1**	1.0	1.3	0	1	3	**1.5**	1.3	1.7
EP	2	4	7	**4.3**	3.7	4.9	0	2	4	**1.8**	1.6	2.0	1	2	4	**2.2**	1.9	2.4
H	7	9	10	**8.8**	8.4	9.1	0	2	4	**2.0**	1.7	2.2	0	3	6	**2.9**	2.6	3.2
PB(i)	1	2	4	**2.5**	2.1	2.9	0	1	2	**0.8**	0.7	1.0	0	1	3	**1.1**	0.9	1.2
PP	2	4	6	**4.0**	3.5	4.6	0	1	2	**0.8**	0.7	0.9	0	1	3	**1.1**	1.0	1.3
AvP	4	6	7	**5.3**	5.0	5.7	1	2	3	**1.7**	1.5	1.8	1	2	4	**2.1**	2.0	2.3
SCQ	4	7	13	**7.6**	6.4	8.9	1	3	5	**3.0**	2.8	3.3	1	3	6	**3.5**	3.3	3.8

	All Probands (n=1068)						All Siblings (n=1446)						All (n=2514)					
	Q25	*Q50*	*Q75*	*mean $_t$*	*Ci $_{t,low}$*	*Ci $_{t,up}$*	*Q25*	*Q50*	*Q75*	*mean $_t$*	*Ci $_{t,low}$*	*Ci $_{t,up}$*	*Q25*	*Q50*	*Q75*	*mean $_t$*	*Ci $_{t,low}$*	*Ci $_{t,up}$*
CP	3	5	6	**4.6**	4.4	4.8	0	0	1	**1.6**	1.5	1.7	1	3	5	**2.9**	2.7	3.0
EP	2	4	6	**3.7**	3.5	3.9	0	2	4	**1.8**	1.7	2.0	1	3	5	**2.7**	2.5	2.8
H	8	9	10	**8.8**	8.7	8.9	1	3	6	**2.9**	2.7	3.1	2	6	9	**5.9**	5.7	6.1
PB(i)	1	3	5	**3.0**	2.8	3.1	0	1	3	**1.4**	1.3	1.6	1	2	4	**2.1**	2.0	2.2
PP	2	4	6	**3.9**	3.7	4.0	0	1	3	**1.2**	1.0	1.3	0	2	4	**2.2**	2.1	2.4
AvP	4	5	6	**5.2**	5.1	5.3	1	2	4	**2.1**	2.0	2.2	2	4	5	**3.6**	3.5	3.7
SCQ	4	8	12	**7.6**	7.2	8.0	1	3	6	**3.7**	3.5	3.9	2	5	9	**5.2**	5.0	5.4

Teacher ratings

	Male Probands (n=938)						Male Siblings (n=730)						All boys (n=1668)						
	Q25	*Q50*	*Q75*	*mean $_t$*	*Ci $_{t,low}$*	*Ci $_{t,up}$*	*Q25*	*Q50*	*Q75*	*mean $_t$*	*Ci $_{t,low}$*	*Ci $_{t,up}$*	*Q25*	*Q50*	*Q75*	*mean $_t$*	*Ci $_{t,low}$*	*Ci $_{t,up}$*	
CP	1	3	5	**2.9**	2.8	3.1	0	1	3	**1.3**	1.1	1.4	1	2	4	**2.2**	2.1	2.4	***CP***
EP	1	3	4	**2.6**	2.4	2.8	0	1	3	**1.4**	1.3	1.6	1	2	4	**2.1**	2.0	2.2	***EP***
H	7	8	10	**8.3**	8.1	8.5	2	4	7	**4.6**	4.3	4.9	4	7	9	**7.0**	6.8	7.2	***H***
PB(i)	2	4	6	**4.3**	4.1	4.5	1	3	5	**3.1**	2.9	3.3	2	4	6	**3.8**	3.6	4.0	***PB(i)***
PP	1	3	5	**2.8**	2.7	3.0	0	1	3	**1.4**	1.2	1.5	1	2	4	**2.2**	2.0	2.3	***PP***
AvP	3	4	5	**4.2**	4.1	4.3	1	3	4	**2.4**	2.3	2.6	2	4	5	**3.5**	3.4	3.6	***AvP***
SCQ																			

	Female Probands (n=130)						Female Siblings (n=716)						All girls (n=846)						
	Q25	*Q50*	*Q75*	*mean $_t$*	*Ci $_{t,low}$*	*Ci $_{t,up}$*	*Q25*	*Q50*	*Q75*	*mean $_t$*	*Ci $_{t,low}$*	*Ci $_{t,up}$*	*Q25*	*Q50*	*Q75*	*mean $_t$*	*Ci $_{t,low}$*	*Ci $_{t,up}$*	
CP	1	2	4	**2.3**	1.9	2.7	0	0	2	**0.5**	0.3	0.6	0	0	2	**0.7**	0.6	0.8	***CP***
EP	1	3	5	**3.1**	2.6	3.6	0	1	3	**1.3**	1.2	1.5	0	1	3	**1.6**	1.4	1.8	***EP***
H	6	8	9	**7.5**	7.0	7.9	0	2	5	**2.4**	2.1	2.7	1	3	6	**3.2**	2.9	3.5	***H***
PB(i)	1	3	5	**3.2**	2.7	3.8	0	1	4	**1.7**	1.4	1.9	0	2	4	**1.9**	1.7	2.1	***PB(i)***
PP	1	3	4	**2.7**	2.3	3.2	0	1	2	**0.9**	0.7	1.1	0	1	3	**1.2**	1.0	1.3	***PP***
AvP	3	4	5	**3.9**	3.7	4.2	1	2	3	**1.5**	1.4	1.7	1	2	3	**1.9**	1.8	2.1	***AvP***
SCQ																			

	All Probands (n=1068)						All Siblings (n=1446)						All (n=2514)						
	Q25	*Q50*	*Q75*	*mean $_t$*	*Ci $_{t,low}$*	*Ci $_{t,up}$*	*Q25*	*Q50*	*Q75*	*mean $_t$*	*Ci $_{t,low}$*	*Ci $_{t,up}$*	*Q25*	*Q50*	*Q75*	*mean $_t$*	*Ci $_{t,low}$*	*Ci $_{t,up}$*	
CP	1	3	5	**2.9**	2.7	3.0	0	1	2	**0.8**	0.7	0.9	0	2	3	**1.7**	1.5	1.8	***CP***
EP	1	3	4	**2.7**	2.5	2.8	0	1	3	**1.4**	1.3	1.5	0	2	4	**2.0**	1.8	2.1	***EP***
H	7	8	10	**8.2**	8.1	8.4	1	3	6	**3.5**	3.2	3.7	3	6	9	**5.8**	5.7	6.0	***H***
PB(i)	2	4	6	**4.2**	4.0	4.4	1	2	4	**2.4**	2.2	2.5	1	3	5	**3.2**	3.0	3.3	***PB(i)***
PP	1	3	5	**2.8**	2.7	3.0	0	1	3	**1.1**	1.0	1.2	0	2	4	**1.8**	1.7	1.9	***PP***
AvP	3	4	5	**4.2**	4.1	4.3	1	2	3	**2.0**	1.9	2.1	2	3	5	**3.0**	2.9	3.1	***AvP***
SCQ																			

CP SDQ: Conduct Problems
EP SDQ: Emotional Problems
H SDQ: Hyperactivity
PB(i) SDQ: Prosocial Behaviour (inverted)
PP SDQ: Peer Problems
AvP SQQ: Average Problems

SCQ Social Communications Questionnaire

Q25 25th quantile
Q50 Median
Q75 75th quantile
mean $_t$ 20% trimmed mean
Ci $_{t, low}$ 95% Confidence interval for trimmed mean (lower end)
Ci $_{t, up}$ 95% Confidence interval for trimmed mean (upper end)
