# Supplementary material for: The impact of study design and diagnostic approach in a large multi-centre ADHD study: Part 2: Dimensional measures of psychopathology and intelligence
Source: BMC Psychiatry. 2011 Apr 7;11:55. doi: 10.1186/1471-244X-11-55 (PMC3090338; doi:10.1186/1471-244X-11-55)
Supplement: Additional file 7 — Table S3. Quantiles and trimmed means with confidence intervals of intelligence measures. [file 1471-244X-11-55-S7.PDF]

Table S3: IQ (prorated) and subtests

	Male Probands (n=717-738)						Male Siblings (n=480-492)						All boys (n=1197-1231)					
	Q25	*Q50*	*Q75*	*$mean_t$*	*$Ci_{t,low}$*	*$Ci_{t,up}$*	*Q25*	*Q50*	*Q75*	*$mean_t$*	*$Ci_{t,low}$*	*$Ci_{t,up}$*	*Q25*	*Q50*	*Q75*	*$mean_t$*	*$Ci_{t,low}$*	*$Ci_{t,up}$*
IQ	90	102	112	**101.3**	100.1	102.5	95	103	114	**103.8**	102.5	105.1	92	103	113	**102.4**	101.4	103.3
V	8	10	12	**9.80**	9.56	10.02	9	11	12	**10.49**	10.21	10.76	8	10	12	**10.08**	9.91	10.26
S	9	11	13	**10.65**	10.41	10.92	9	11	13	**10.94**	10.66	11.23	9	11	13	**10.77**	10.59	10.97
PC	9	11	13	**10.87**	10.65	11.11	9	11	13	**10.77**	10.49	11.01	9	11	13	**10.83**	10.66	10.99
BD	8	10	12	**9.64**	9.40	9.89	8	10	12	**10.13**	9.88	10.40	8	10	12	**9.85**	9.67	10.02
DS	7	9	11	**8.62**	8.41	8.82	8	10	11	**9.48**	9.21	9.77	7	9	11	**8.95**	8.79	9.11

	Female Probands (n=97-104)						Female Siblings (n=493-509)						All girls (n=590-1231)					
	Q25	*Q50*	*Q75*	*$mean_t$*	*$Ci_{t,low}$*	*$Ci_{t,up}$*	*Q25*	*Q50*	*Q75*	*$mean_t$*	*$Ci_{t,low}$*	*$Ci_{t,up}$*	*Q25*	*Q50*	*Q75*	*$mean_t$*	*$Ci_{t,low}$*	*$Ci_{t,up}$*
IQ	90	98	106	**97.9**	95.0	100.8	93	102	112	**101.7**	100.4	103.1	92	102	110	**101.1**	99.9	102.3
V	7	9	12	**9.27**	8.61	9.94	8	10	12	**9.98**	9.74	10.22	8	10	12	**9.88**	9.62	10.10
S	8	10	11	**9.81**	9.36	10.32	9	11	13	**10.80**	10.54	11.05	9	11	12	**10.64**	10.37	10.85
PC	8	10	12	**10.24**	9.69	10.78	8	10	12	**10.34**	10.07	10.62	8	10	12	**10.32**	10.06	10.57
BD	8	9	11	**9.39**	8.88	9.91	8	10	12	**9.85**	9.56	10.10	8	10	12	**9.77**	9.52	9.99
DS	7	9	11	**9.11**	8.63	9.70	8	10	12	**9.74**	9.45	9.96	8	10	11	**9.63**	9.40	9.86

	All Probands (n=814-842)						All Siblings (n=974-1002)						All (n=1788-1844)					
	Q25	*Q50*	*Q75*	*$mean_t$*	*$Ci_{t,low}$*	*$Ci_{t,up}$*	*Q25*	*Q50*	*Q75*	*$mean_t$*	*$Ci_{t,low}$*	*$Ci_{t,up}$*	*Q25*	*Q50*	*Q75*	*$mean_t$*	*$Ci_{t,low}$*	*$Ci_{t,up}$*
IQ	90	101	111	**100.9**	99.7	102.0	94	103	112	**102.8**	101.8	103.7	92	102	112	**101.9**	101.2	102.6
V	8	10	12	**9.73**	9.52	9.94	8	10	12	**10.20**	10.02	10.40	8	10	12	**10.01**	9.87	10.14
S	8	10	13	**10.53**	10.32	10.77	9	11	13	**10.87**	10.68	11.04	9	11	13	**10.73**	10.58	10.87
PC	9	11	13	**10.79**	10.57	10.99	9	11	13	**10.55**	10.35	10.75	9	11	13	**10.66**	10.51	10.80
BD	8	10	12	**9.61**	9.38	9.81	8	10	12	**9.99**	9.81	10.17	8	10	12	**9.82**	9.68	9.96
DS	7	9	11	**8.69**	8.48	8.87	8	10	11	**9.61**	9.42	9.80	7	9	11	**9.16**	9.03	9.31

IQ	Prorated IQ (see Methods)	*Q25*	25th quantile
V	Vocabulary	*Q50*	Median
S	Simliarities	*Q75*	75th quantile
PC	Picture Completion	*$mean_t$*	20% trimmed mean
BD	Block Design	*$Ci_{t, low}$*	95% CI for trimmed mean (lower end)
DS	Digit Span	*$Ci_{t, up}$*	95% CI for trimmed mean (upper end)
