# Supplementary material for: The impact of study design and diagnostic approach in a large multi-centre ADHD study: Part 2: Dimensional measures of psychopathology and intelligence
Source: BMC Psychiatry. 2011 Apr 7;11:55. doi: 10.1186/1471-244X-11-55 (PMC3090338; doi:10.1186/1471-244X-11-55)
Supplement: Additional file 8 — Figure S5. Post-hoc comparisons of the prorated IQ. [file 1471-244X-11-55-S8.PDF]

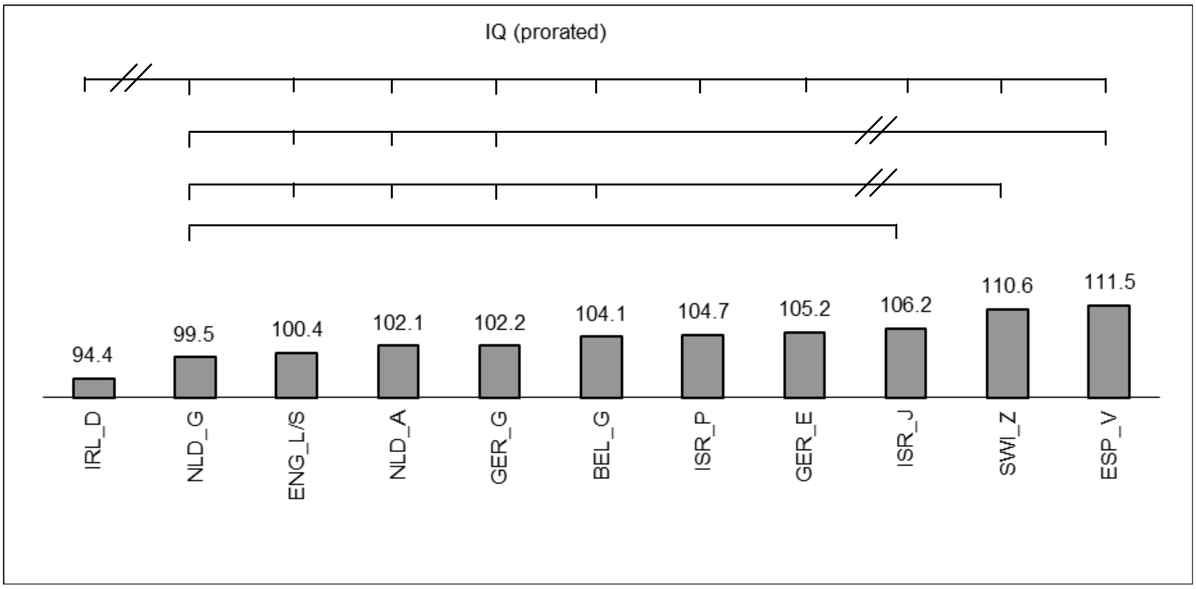

Figure S5
Mean IQ across centres with significant post hoc comparisons (5% family wise error rate).
Notes: Small vertical lines on the left of each double slash sign indicate centres with lower IQ compared to the centres indicated by the vertical lines on the right of the double slash sign. Abbreviations of the centres are explained in the text.
